# Metagenomics revealing molecular profiling of community structure and metabolic pathways in natural hot springs of the Sikkim Himalaya

**DOI:** 10.1186/s12866-020-01923-3

**Published:** 2020-08-10

**Authors:** Nitish Sharma, Jitesh Kumar, Md. Minhajul Abedin, Dinabandhu Sahoo, Ashok Pandey, Amit K. Rai, Sudhir P. Singh

**Affiliations:** 1 Center of Innovative and Applied Bioprocessing (DBT-CIAB), SAS Nagar, Mohali India; 2 Department of Biotechnology, Panjab University, Chandigarh, India; 3 Institute of Bioresources and Sustainable Development, Sikkim Centre, Tadong, Gangtok, India; 4 CSIR-Indian Institute of Toxicology Research, Lucknow, India

**Keywords:** Hot springs, Metagenomics, Taxonomic profiling, Functional potential, Antibiotic resistance, CAZymes, Glycosyltransferases

## Abstract

**Background:**

Himalaya is an ecologically pristine environment. The geo-tectonic activities have shaped various environmental niches with diverse microbial populations throughout the Himalayan biosphere region. Albeit, limited information is available in terms of molecular insights into the microbiome, including the uncultured microbes, of the Himalayan habitat. Hence, a vast majority of genomic resources are still under-explored from this region. Metagenome analysis has simplified the extensive in-depth exploration of diverse habitats. In the present study, the culture-independent whole metagenome sequencing methodology was employed for microbial diversity exploration and identification of genes involved in various metabolic pathways in two geothermal springs located at different altitudes in the Sikkim Himalaya.

**Results:**

The two hot springs, Polok and Reshi, have distinct abiotic conditions. The average temperature of Polok and Reshi was recorded to be 62 °C and 43 °C, respectively. Both the aquatic habitats have alkaline geochemistry with pH in the range of 7–8. Community profile analysis revealed genomic evidence of plentiful bacteria, with a minute fraction of the archaeal population in hot water reservoirs of Polok and Reshi hot spring. Mesophilic microbes belonging to Proteobacteria and Firmicutes phyla were predominant at both the sites. Polok exhibited an extravagant representation of Chloroflexi, Deinococcus-Thermus, Aquificae, and Thermotogae. Metabolic potential analysis depicted orthologous genes associated with sulfur, nitrogen, and methane metabolism, contributed by the microflora in the hydrothermal system. The genomic information of many novel carbohydrate-transforming enzymes was deciphered in the metagenomic description. Further, the genomic capacity of antimicrobial biomolecules and antibiotic resistance were discerned.

**Conclusion:**

The study provided comprehensive molecular information about the microbial treasury as well as the metabolic features of the two geothermal sites. The thermal aquatic niches were found a potential bioresource of biocatalyst systems for biomass-processing. Overall, this study provides the whole metagenome based insights into the taxonomic and functional profiles of Polok and Reshi hot springs of the Sikkim Himalaya. The study generated a wealth of genomic data that can be explored for the discovery and characterization of novel genes encoding proteins of industrial importance.

## Background

The high-throughput sequence analysis of the genetic material has led to the cultivation-independent and comprehensive investigation of microbial and functional diversity of the ecological niches. The metagenomic study is increasingly being performed to resolve and characterize the microbial signatures and functional potential of the natural habitats. The analysis of culture-independent metagenome sequence data is helpful in elucidating the association among taxonomic composition, their functioning, and environmental feature [1]. Metagenomics can exert a significant impact on understanding the microbial niches of the extreme habitats, such as hot springs, glacial lakes, hydrothermal vents, etc., which are often found in isolated places. Further, the bioinformatic analysis of the assembled-metagenomic information generated from the extreme environmental samples, such as hot-springs, has led to the discovery of novel genes encoding desirable biocatalysts of societal importance [2–5].

Himalaya is a treasure of unexplored microbial wealth in the ecological niches of characteristic temperature and surrounding rocks composition. Sikkim is a Himalayan state in the north-east region of the Indian union territory, which resides in the vicinity of the tectonically active domain of the eastern Himalayas [6]. The Himalayan geothermal region has several hot-water ecosystems, which were naturally created millions of years ago by tectonic activities. In Sikkim, the ancient thermal springs, locally known as Tatopani or Tsha-Chu, are of religious, medical, and aesthetic values. The therapeutic significance of geothermal springs is documented globally in many reports. These hot-springs are the rich sources of ores of precious elements, other chemicals, as well as a wealth of microbial diversity. The microflora of the hot springs is directly dependent upon the physicochemical characteristics of the site, i.e., pH, redox potential, temperature, mineral and other chemical profiles [7]. Here, it is essential to note that the chemical composition of the thermal reservoirs depends on the chemical nature of the surrounding rock materials and their chemical interaction with the water [8]. Metagenomic investigation can divulge the molecular ecological features, revealing the vast and diverse genetic pool of these natural habitats. However, hardly any study has been conducted to explore the whole-metagenome based in-depth molecular profiling of taxonomic diversity and metabolic features of the remotely located geothermal sites in the Himalayan region.

The microbiota of the thermal aquatic habitats of high elevation in the Sikkim region is underexplored at the genomic level. The study of microbial diversity in the samples from the Yumthang hot-spring of the Sikkim region revealed information about several thermo-adapted bacteria at this site [9, 10]. Recently, 16S rRNA gene amplicon sequencing has been performed, investigating the microbial ecological details of Polok and Borong hot-springs in Sikkim [11]. However, the whole metagenomic perusals of the extreme ecosystems of this region, exploring the biosynthetic applications of their genetic resources, are quite limited. The biocatalysts encoded in the genome of thermophilic microorganisms residing in the harsh natural sources often exhibit inherent desirable properties of high thermal stability, resistance to organic solvent, and long-term storage. The comprehensive metagenomic investigation of the ecological niches facilitates taxonomic binning, and the assessment of the metabolic and functional potential in the habitat, deciphering the molecular basis of the unique properties of the environmental sites.

In the present work, we analyzed the whole metagenome sequences derived from two remote hot springs of the Sikkim Himalayas, Polok, and Reshi. This investigation aimed to study the genomic features of these sites and decipher the microbial diversity, metabolic and functional profiling of the thermal aquatic bioresource, generating a metagenomic wealth for the discovery and characterization of novel genes encoding biocatalysts of industrial potential.

## Results

### Site description

**Fig. 1:**
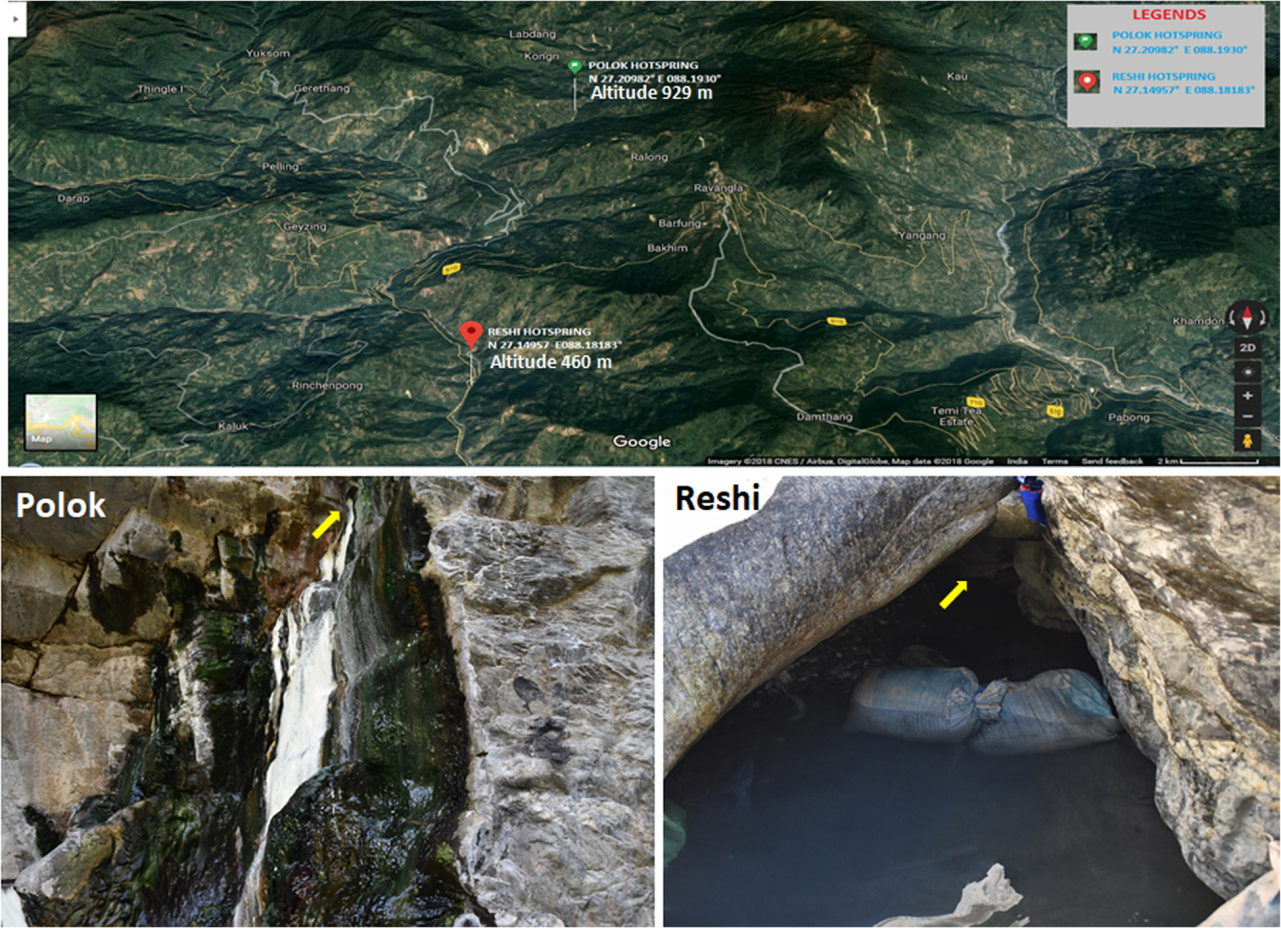
Sampling sites and geographical positioning of Polok and Reshi hot springs. The geographical positions of the sites have been shown by using the Google Maps, 2020

Polok and Reshi hot springs are located at different altitudes near the river ‘Rangit’. Polok hot spring is situated at the altitude of 929 m, with coordinates of 27.3504235° N, and 88.3203041° E. The altitude of the Reshi hot spring was measured as 460 m, with coordinates of 27.2415603° N, and 88.2989453° E (Fig. 1) [12]. The sampling has been done at the place where the hot water oozes at the surface of the Earth. The average temperature and pH of the hot spring water at the sampling site were recorded to be 62 °C and pH 8.0 in Polok, and 43 °C and pH 7.5 in Reshi.

### Metagenome sequencing and taxonomic annotation

Metagenomic DNA was extracted from the water samples collected from Polok and Reshi. Whole metagenome sequencing generated about 18.4 and 19.1 million high-quality paired ends (PE) reads from Polok and Reshi samples, respectively. These reads were assembled into about 149.4 and 121.7 thousand scaffolds with an average size of 905 and 877 bp, and the N50 value of about 1207 bp and 1202 bp in Polok and Reshi metagenomes, respectively. A total of 110,729 and 106,626 ORFs were predicted from the assembled data of Polok and Reshi, with the average size of 797 and 702 bp, and minimum size of 402 and 351 bp, respectively (Table 1).

**Table 1:** Metagenome sequencing and assembly statistics of Polok and Reshi hot springs

Statistics	Polok	Reshi
Number of reads	18,489,382	19,152,814
Number of scaffolds	149,440	121,736
Average scaffold size (bp)	905.6	877.79
N50 value of Scaffold	1207	1202
Total ORFs predicted	110,729	106,626
Average size of the ORFs	797.45	701.79
ORFs mapped to COG	77,700	78,212
ORFs mapped to KEGG database	98,932	94,901
ORFs mapped to GO database	109,705	110,114
ORFs mapped to Pfam database	84,173	84,517
ORFs mapped to FigFam database	51,509	51,626
ORFs mapped to nr database	110,170	103,006
ORFs mapped to CAZy database	18,374	9873

**Fig. 2:**
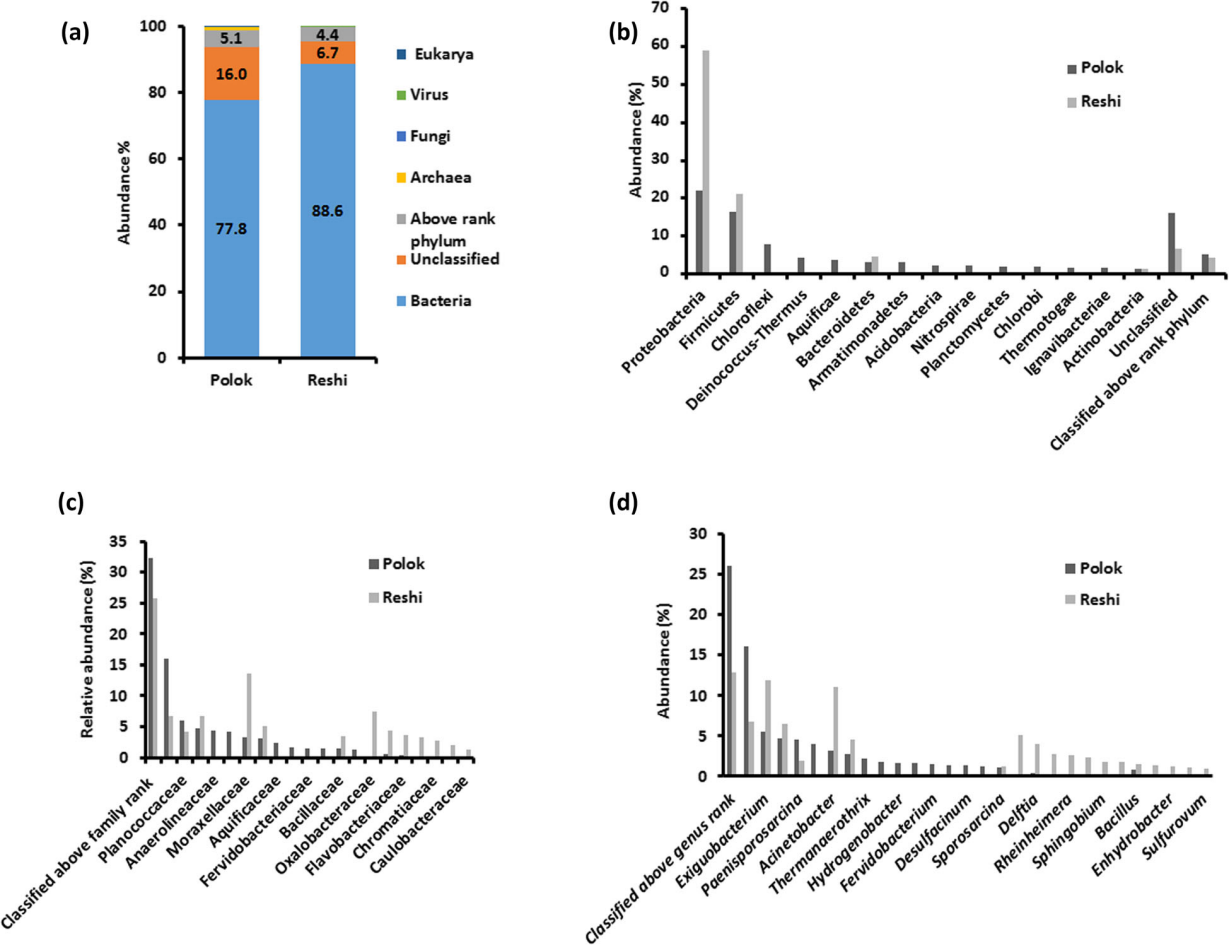
An overview of taxonomic profile in Polok and Reshi hot springs. **a** Domain level taxonomy, **b** phylum level taxonomy, **c** family level taxonomy, **d** genus level taxonomy

The taxonomic binning of the predicted ORFs revealed the dominance of bacterial organisms, showing an abundance of 77.8 and 88.6% in Polok and Reshi, respectively (Fig. 2a; Table S1). About 0.95 and 0.11% of the total ORFs were accounted for the distribution of the archaeal communities in Polok and Reshi, respectively. The representation of viruses was very less in both the samples (~ 0.03%). The percentage of ORFs with unassigned taxa and classified above the rank phylum were 16 and 5% in Polok, and 6.7 and 4.4% in Reshi metagenomes, respectively.

The predominant bacterial phyla identified in Polok sample were Proteobacteria (22.06%), Firmicutes (16.36%), Chloroflexi (7.7%), and Deinococcus-Thermus (4.2%). Reshi metagenome exhibited the highest abundance of Proteobacteria (59%), Firmicutes (21%), and Bacteroidetes (4.4%) (Fig. 2b; Table S2). At the family level, Planococcaceae (6%), Pseudomonadaceae (4.74%), Anaerolineaceae (4.45%), Thermaceae (4.15%), and Moraxellaceae (3.21%) were dominant in Polok sample. In Reshi, family Moraxellaceae was accounted for 13.56% of the mapped ORFs, followed by Oxalobacteraceae (7.52%), Pseudomonadaceae (6.65%), and Xanthomonadaceae (5.12%) (Fig. 2c; Table S3). The ORFs exhibited an association with a total of 1954 genera, out of which 1210 were common in both the metagenomic samples, whereas 526 and 217 genera were unique to Polok and Reshi, respectively. *Exiguobacterium* (5.47%) was the most abundant genus in Polok sample, followed by *Pseudomonas* (4.74%)*, Paenisporosarcina* (4.54%), *Thermus* (3.94%), *Acinetobacter* (3.19%), *Stenotrophomonas* (2.71%), *Thermanaerothrix* (2.24%), and *Thermoanaerobaculum* (1.74%), etc. In the case of Reshi, the highest abundance was observed for *Exiguobacterium* (11.85%) and *Acinetobacter* (11.08%), followed by *Pseudomonas* (6.52%), *Massilia* (5.17%), *Stenotrophomonas* (4.6%), etc. (Fig. 2d; Table S4).

The sequences belonging to thermophilic genera like *Thermus* (3.94%), *Thermanaerothrix* (2.24%), *Hydrogenobacter* (1.65%), *Anaerolinea* (1.63%), *Fervidobacterium* (1.51%), *Sulfurihydrogenibium* (1.28%), were found only in Polok. The composition of mesophilic microbes was also distinct in Polok and Reshi. Reshi hot spring evinced a relatively more diverse proteobacterial community structure, comprised of alpha-proteobacteria (*Sphingobium*, *Rhizobium*, *Sphingopyxis*, *Methylocapsa*, and *Brevundimonas*), beta-proteobacteria (*Massilia*, *Thiomonas*, *Janthinobacterium*, and *Duganella*), gamma-proteobacteria (*Rheinheimera*, *Moraxella*, and *Enhydrobacter*), epsilonproteobacteria (*Sulfovorum*), and deltaproteobacteria (*Desulfatirhabdium*). Polok displayed the presence of four common (*Acenatobacter*, *Pseudomonas*, *Delftia*, and *Stenotrophomonas*), and three disparate (*Ochrobactrum Desulfacinum*, and *Desulfobacca*) proteobacterial genera (Table S4).

The taxonomic distribution at species level indicated the metagenomic affiliation of 5396 species in Polok and 6382 species in Reshi, with 3118 species common in both the hot spring samples (Fig. S1A; Table S5). Both the hot springs manifested dominance of mesophiles, followed by thermophiles, and scant presence of psychrophiles (Fig. S1B; Table S6). Polok metagenome conveyed the abundance of *Paenisporosarcina* sp. HGH0030 (3.25%), *Stenotrophomonas maltophilia* (2.26%), *Thermanaerothrix daxensis* (2.24%), *Exiguobacterium pavilionensis* (2.11%), *Thermoanaerobaculum aquaticum* (1.74%), and *Hydrogenobacter thermophilus* (1.65%). The prevalent species in Reshi metagenome were *Exiguobacterium pavilionensis* (4.17%), *Stenotrophomonas maltophilia* (3.84%), *Cloacibacterium normanense* (2.72%), *Pseudomonas mosselii* (2.4%), *Acinetobacter kookii* (2.26%), and *Moraxella osloensis* (2.15%). Among the archaeal community, substantial representation of phylum Euryarchaeota (0.85%) was observed in Polok. Furthermore, a fairly good percentage of ORFs were accredited with the unclassified sequences (16.05% in Polok, and 6.72% in Reshi), and classified above rank species (21.51% in Polok, and 28.86% in Reshi).

### Microbial diversity and co-occurrence

The Pearson product-moment correlation among the variables (phyla) was ranged between − 1 and + 1, indicating the strength of the linear relationship among them. The *P*-value for the correlated pairs was statistically significant, i.e., P-value < 0.05 and with 95.0% confidence. Proteobacteria, Firmicutes, and Bacteroidetes showed a positive correlation with each other. On the contrary, Proteobacteria, Firmicutes, and Bacteroidetes maintained a negative relation with the other 11 phyla, e.g., Chloroflexi, Deinococcus-Thermus, Thermotogae, Aquificae, Armatimonadetes, Acidobacteria, Nitrospirae, Planctomycetes, Chlorobi, Actinobacteria and Ignavibacteriae (Fig. S2; Table S7). Interestingly, these 11 phyla were positively correlated with one another. The statistical significance of the relative abundance of the species was tested with respect to one another in the range of − 1 to +1. The moderate intersection of the species indicated the genomic presence of the diverse microbial flora in Polok and Reshi (Fig. S3).

SHE (S: species richness, H: Shannon-Wiener evenness index and E: evenness as measured by Shannon-Wiener index) analysis inferred substantially high species richness (S) at the diversity scale of 2.8 to 3.0 (Fig. S4). However, evenness (E) of the species was found quite low, manifesting the unequal distribution of the species in Polok and Reshi. H index of 3.5 to 4 indicated the existence of a diverse microbial profile in both the hot water reservoirs, with a low level of species similarity between them. The values of global β-diversity indices were ranged from 0.120 to 0.4. The low β-diversity index further corroborates low species similarity between the two examined habitats (Table 2).

**Table 2:** The measures of beta-diversity, representing species richness in Polok and Reshi

Beta diversity criteria	value
Routledge	0.12
Williams	0.28
Whittaker	0.4
Harrison	0.4
Wilson-Shmida	0.4
Mourelle	0.4
Harrison 2	0.4

In PCA analysis, the PC1 and PC2 axes represented 68.257 and 31.743% variance, with eigenvalues of 1.859, and 0.864, respectively. All the species were clustered in four different coordinates of the graph at 95% confidence level. The clustering of the species was done in accordance with their relative abundance in the hot springs. Highly abundant species were grouped in the positive coordinate of the respective hot spring, whereas the species in scarce were grouped in the negative coordinate (Fig. S5; Table S8).

### Functional potential analysis

To infer the functional potential of the hot spring samples, the predicted ORFs were aligned against COG and KEGG databases. A total of 77,700 and 78,212 predicted genes or ORFs from Polok and Reshi metagenomes were mapped to 3486 and 3444 COG functions, respectively. COG categorization depicted the predominance of the functions related to amino acid transport and metabolism, cell wall/membrane biogenesis, replication, recombination and repair, energy production and conversion, inorganic ion transport and metabolism, carbohydrate transport and metabolism in the metagenomic data of both the samples (Table S9). In particular, the genes related to glycosyltransferase, permeases, signal transduction histidine kinase, dehydrogenases, methyltransferases, and transcriptional regulators, etc., were found abundant in both Polok and Reshi metagenomes. Interestingly, a COG function, outer membrane receptor proteins for iron transport, was highly represented in the Reshi sample, as compared to Polok.

KEGG is a unique pathway repository that helps to decipher the putative metabolic pathways associated with an organism or the metagenome of a particular niche. KEGG gene annotation mapped a total of 98,932 (Polok), and 94,901 (Reshi) predicted genes, identifying 4576 and 4519 KEGG Orthology (KO) terms, respectively (Table S10). The most represented KEGG term was two-component system. More than 5000 ORFs were mapped against the 169 unique KO id’s belonging to the two-component system pathways in both Polok and Reshi hot springs. Among them, the ORFs related to the protein family of histidine kinase PdtaS were in abundance, followed by Nitrogen regulatory protein (NtrC) and the DNA-binding protein OmpR families. The other abundant KO terms were F420H (2)-dependent quinone reductase, L-malate glycosyltransferase, ovochymase, methyltransferase-like protein-6, iron complex outer-membrane receptor protein, chemotaxis family protein, N-acetyltransferase, and hydrophobic/amphiphilic HAE1 family exporter-1. The representation of KO ids associated with iron transport and heavy-metal exporter and chemotaxis family proteins was relatively higher in Reshi metagenome. In contrast, the predominance of hetero-disulfide reductase subunits was observed in Polok. KEGG pathway analysis uncovered different metabolic pathways, which are crucial for not only the survival of the resident microbes but also credit beneficial impacts on the commuters visiting these sites.

### Metabolic pathway gene pool

#### Nitrogen metabolism

Polok and Reshi hot springs are known to be moderately rich in nitrogen content in the form of extracellular nitrate (NaNO_3_) [13]. Microbes utilize nitrogen in both assimilatory as well as dissimilatory ways by reducing nitrogen into ammonia, followed by its subsequent utilization in amino acid biosynthesis pathways. A total of 1489 ORFs (758 from Polok, and 731 from Reshi) were mapped on 36 KO ids related to the nitrogen metabolism. Microbes have a complex network of membrane transporter proteins, which facilitate the diffusion of inorganic nitrogen into the intracellular environment. In both the metagenomes, genes related to nitrate/nitrite porter (NNP) family, within the major facilitator superfamily (MFS), were identified that could be involved in the uptake of nitrogen oxyanion, i.e., nitrate and nitrite. The intracellular nitrate gets converted to nitrite by the catalytic actions of nitrate reductase. In the metagenomes, putative genes were identified for dissimilatory (narG EC: 1.7.5.1; narH EC: 1.7.5.1; narI EC: 1.7.5.1; napA EC: 1.7.99), and assimilatory (nasA EC: 1.7.99; nasB EC: 1.7.99; narB EC: 1.7.7.2) nitrate reduction (Fig. S6; Table S11). When the intracellular concentration of nitrite exceeds, it may be converted into nitrate by narG (EC: 1.7.5.1) and/or narH (EC: 1.7.5.1), which displays both the activities of nitrate reductase and nitrite oxidoreductase. Inside the cell, the imported nitrite has two fates, either it gets reduced to ammonia, or it may undergo denitrification to produce nitrogen. In the metagenome, putative genes were identified for both the pathways. The nitrite reductase enzymes, nirB (EC: 1.7.1.15), and nirD (EC: 1.7.1.15), and nrfA (EC: 1.7.2.2) were detected, which participate in the conversion of nitrite to ammonia. Intracellular nitrite can also be diverted into the nitrate-nitrite-nitric oxide pathway, especially under hypoxic conditions, by the enzyme nirK; Nitric oxide-forming nitrite reductase (EC: 1.7.2.1). Nitric oxide can be reduced to nitrous oxide by norB (EC: 1.7.2.5) and norC enzymes. The enzyme nosZ (EC: 1.7.2.4) further reduces nitrous oxide into nitrogen. NifD (EC: 1.18.6.1), nifK (EC: 1.18.6.1), and nifH enzymes catalyze the utilization of nitrogen into ammonia formation. The denitrification related genes, involved in nitrate-nitrite-nitric oxide, and nitrous oxide to nitrogen biosynthetic route, were more prominent in Polok as compared to Reshi. The other genes involved in ammonia biosynthesis from the precursors like nitrile (nitrilase EC: 3.5.5.1), formamide (formamidase EC: 3.5.1.49), and hydroxylamine (hydroxylamine reductase EC: 1.7.99.1) were also identified in the metagenomes. Moreover, genes for the utilization of ammoniacynate and L-glutamate biosynthesis were detected. Glutamate biosynthesis was prominent in both the metagenomes. The presence of phylum Nitrospirae in Polok and Reshi corroborates the assimilatory and dissimilatory nitrate reduction by the microbes in the hot springs for the energy-yielding processes [14].

#### Sulfur metabolism

Annotation of ORFs (571 from Polok, and 610 from Reshi) with orthologous KO terms (44) inferred the genomic information about the biocatalysts catalyzing the reduction of sulfate to sulfide in both the metagenomic samples (Fig. S7; Table S12). The putative genes involved in the transformation of sulfate to sulfite via the intermediate compounds, adenosine 5′-phosphosulfate (APS) and 3′-Phosphoadenosine-5′-phosphosulfate (PAPS), were identified. The enzymes, sulfate adenylyltransferase (sat EC: 2.7.7.4; cysD EC: 2.7.7.4; cysN EC: 2.7.7.4) and 3′-phosphoadenosine 5′-phosphosulfate synthase (PAPSS EC: 2.7.7.4; EC: 2.7.1.25), catalyze the transformation of sulfate to APS, whereas, adenylylsulfate kinase (cysC EC: 2.7.1.25) and PAPSS catalyze the conversion of APS to PAPS. Phosphoadenosine phosphosulfate reductase (cysH EC: 1.8.4.8 1.8.4.10) transforms PAPS to sulphite. Sulfite is further converted into sulfide by sulfite reductase (cysJ EC: 1.8.1.2; cysI EC: 1.8.1.2). Polok hot spring displayed the presence of *Sulfurihydrogenibium azorense* and *Desulfacinum infernum*, which are sulfur utilizing microorganisms. Reshi also had the genomic indication of sulfur utilizers viz., *Sulfurovum* sp., and *Desulfatirhabdium butyrativorans*.

#### Methane metabolism

**Fig. 3:**
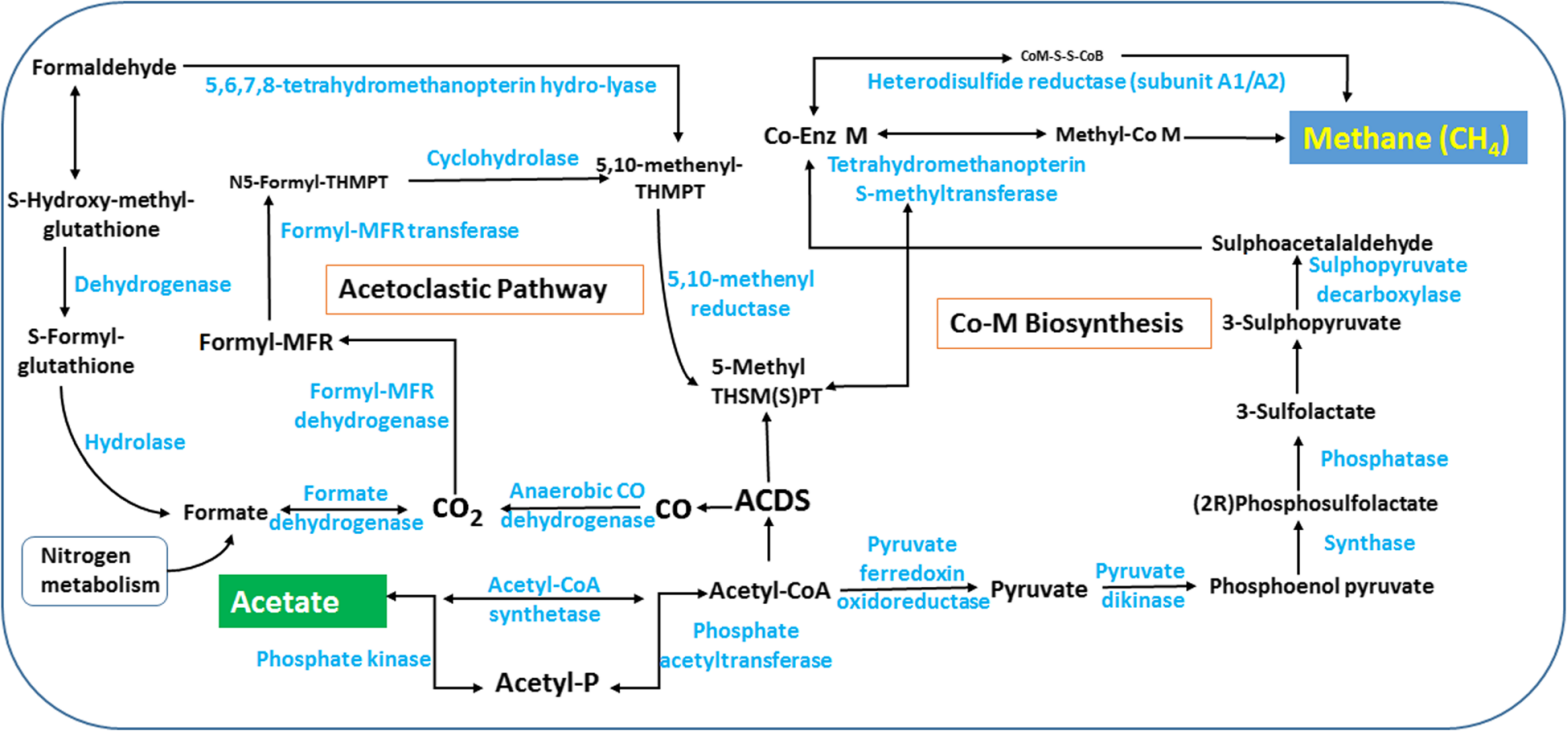
Metagenomic genes mapped to methane metabolism pathway

Methane is one of the potent greenhouse gas on Earth. From the early ages of Earth, this gas is regulating the environment in combination with other gases. In KEGG Orthology, a total of 1715 and 1129 ORFs were found, representing 44 KO ids involved in methane metabolism in Polok and Reshi hot springs, respectively (Table S13). The ORFs belonging to acetate to methane conversion were identified in Polok and Reshi hot springs (Fig. 3). In the methane metabolic pathway, Acetyl-CoA synthetase (EC: 6.2.1.1) catalyzes the conversion of acetate into Acetyl-CoA. Alternatively, acetate may undergo phosphorylation and de-phosphorylation by acetate phosphate transferase (EC: 2.3.1.8) and phosphate kinase (EC: 2.7.2.1), respectively. Acetyl-CoA is transformed to CO, and then CO_2_ by enzymatic actions of decarbonylase (EC: 2.1.1.245; EC: 1.2.7.4), and anaerobic CO dehydrogenase (EC: 1.2.7.4). Then, CO_2_ enters the series of methanogenic steps, making CoM and CoB, which form methane by the catalytic reactions led by MCR enzymes, i.e., methyl CoM reductase enzyme subunits A2 (EC: 1.8.7.3), B2 (EC: 1.8.7.3), and C2 (EC: 1.8.7.3). The MCR enzyme complex catalyzes the last step of methanogenesis and the first step of methanotrophy by reducing CoM/ CoB/ CoM-S-S-CoB complex into the methyl CoM, and finally into methane. Then, methane could be utilized and converted into formaldehyde by methanotrophs. Furthermore, Acetyl-CoA can directly be converted into methyl-TH(S) PT by the enzyme decarbonylase (EC: 2.1.1.245; EC: 1.2.7.4), followed by the action of CoM reductases, synthesizing Co-enzyme M and further into methane. Acetyl-CoA is also involved in the biosynthesis of the CoM via phosphoenolpyruvate pathway. Interestingly, ORFs related to formate and formaldehyde pathways were also detected, which could be involved in CO_2_ reduction and generation of methane (Fig. 3). Polok metagenome exhibited an abundance of *Methanosaeta concilii*, which is an anaerobic acetoclastic archaea, utilizing acetate as the substrate for their growth and perform reduction of CO_2_ to methane. Apart from the methanogens, Polok showed the presence of methanotrophic organisms, such as *Chloroflexus* sp. and *Roseiflexus* sp., which oxidize formaldehyde into CO_2_ using formate dehydrogenase enzyme [15]. Reshi also exhibited the genomic evidence of methanogens (*Methylobacterium populi*) and methanotrophs viz., *Methylocapsa aurea*, *Methylobacterium populi*, and *Methylocapsa acidiphila*.

#### Antibiotic biosynthetic pathways

**Fig. 4:**
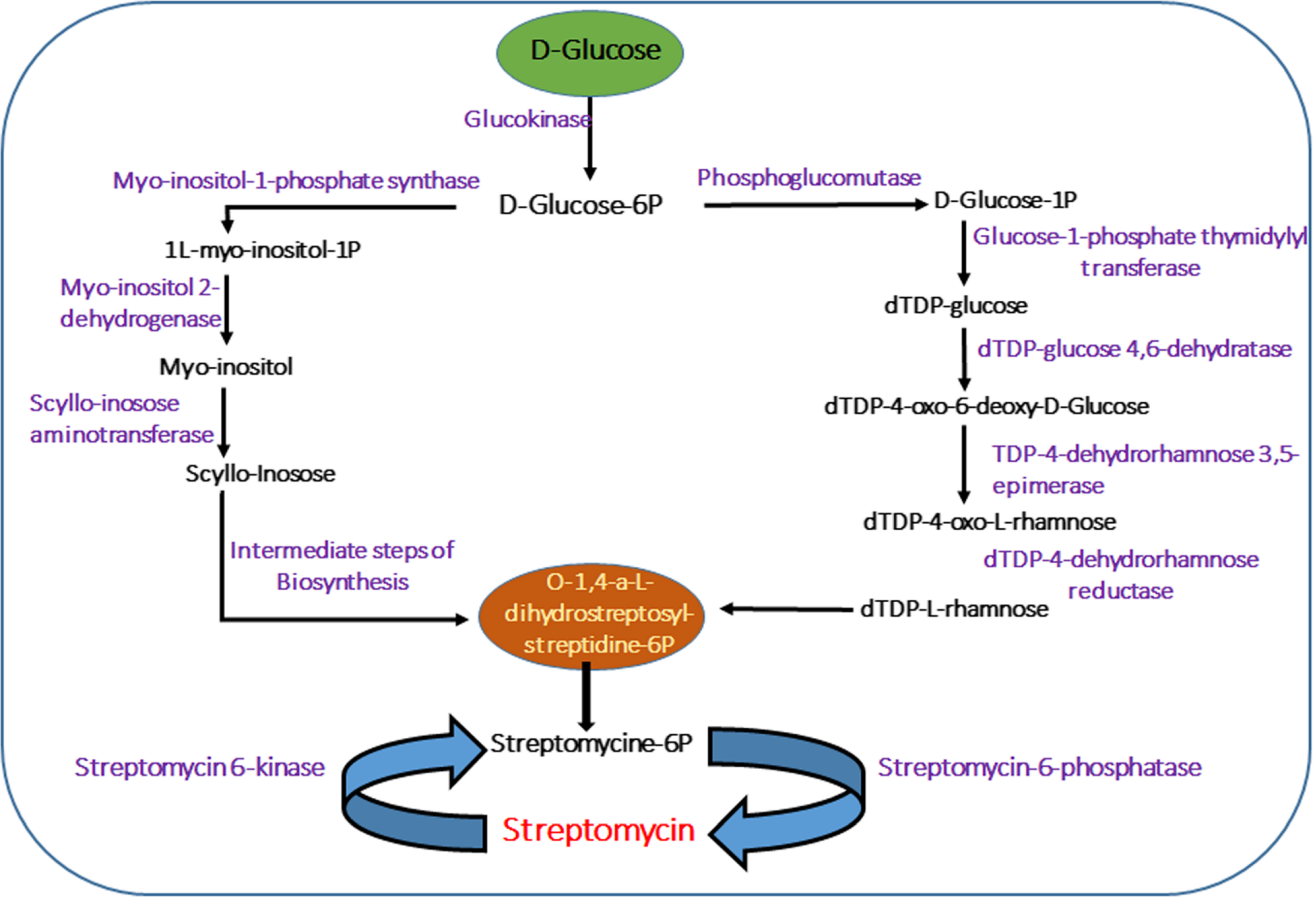
Metagenomic genes mapped to streptomycin biosynthetic pathway

The microbial life in hot springs secretes numerous biomolecules of human health significance, such as antimicrobial compounds. The mapping of metagenomic sequences on metabolic pathways evidenced KO ids belonging to the biosynthesis of different antibiotics viz. streptomycin, monobactam antibiotics, vancomycin, penicillin, and cephalosporin (Table S14). The inception of streptomycin biosynthesis occurs when D-Glucose gets converted into D-glucose-6-phosphate by the enzyme glk; glucokinase (EC: 2.7.1.2), which acts as the precursor molecule. Figure 4 represents the streptomycin biosynthetic pathway, for which most of the orthologous genes were detected in the hot spring metagenome (Table S14). D-glucose-6-phosphate undergoes streptomycin biosynthesis via myo-inositol or L-rhamnose biosynthetic routes. Phosphoglucomutase (EC: 5.4.2.2) converts D-glucose-6-phosphate to D-glucose-1-phosphate. D-glucose-1-phosphate thymidylyltransferase (EC: 2.7.7.24) catalyzes the conversion of D-glucose-1-phosphate to deoxy-thymidinediphosphate-glucose dTDP-glucose. The latter is converted into dTDP-4-oxo-6-deoxy-D-glucose, and then dTDP-4-oxo-L-rhamnose by the catalytic actions of D-TDP-glucose-4, 6-dehydratase (EC: 4.2.1.46), and dTDP-4-dehydrorhamnose 3, 5-epimerase (EC: 5.1.3.13), respectively. The dTDP-l-L-rhamnose is subsequently transformed into streptomycin via the intermediates- dTDP-L-dihydrostreptose, O-1, 4-α-L-dihydro-streptosyl-streptidine-6-phosphate, Dihydrostreptomycin-6 phosphate, and streptomycin 6-phosphate. Alternatively, myo-inositol-1-phosphate synthase (EC: 5.5.1.4) can convert D-glucose-6-phosphate into L-myo-inositol-1-phosphate, which is subsequently transformed into streptomycin through multiple catalytic steps involving myo-inositol 2-dehydrogenase / D-chiro-inositol 1-dehydrogenase (EC: 1.1.1.18; EC:1.1.1.369), L-glutamine:scyllo-inosose aminotransferase (EC: 2.6.1.50), streptomycin 6-kinase (EC: 2.7.1.72), etc. Actinobacteria, which exhibit the characteristics of both bacteria and fungus, are known to produce antibiotics in diverse environmental conditions. They could be the potential source of antibiotic biosynthesis in the Himalayan geothermal habitats.

#### Antibiotic resistance genes

Putative genes were identified for β-lactamases (111 ORFs in Reshi and 83 ORFs in Polok), which inhibit the action of β-lactam antibiotics such as penicillin, cephalosporin, amoxicillin, etc. There was a substantial representation of genes that putatively take part in the biosynthesis and stabilization of the cell wall in Polok and Reshi (Fig. S8; Table S15 A). A total of 541 and 605 ORFs were mapped to β-lactam resistance in Polok and Reshi hot springs, respectively. Among them, the maximum number of ORFs was affiliated to penicillin-binding proteins. Further, a reasonably good number of ORFs were mapped to class C proteins of beta-lactamases, which shows a broad spectrum β-lactams inhibition, providing immunity to the host microbes against many lethal drugs [16]. Different species of *Pseudomonas*, found in Polok (*Pseudomonas fluorescens*, *Pseudomonas* sp. CBZ-4, and *Pseudomonas* sp. S1E40) and Reshi (*Pseudomonas mosselii*, *Pseudomonas putida*, and *Pseudomonas aeruginosa*), could be the source of β-lactamases.

**Fig. 5:**
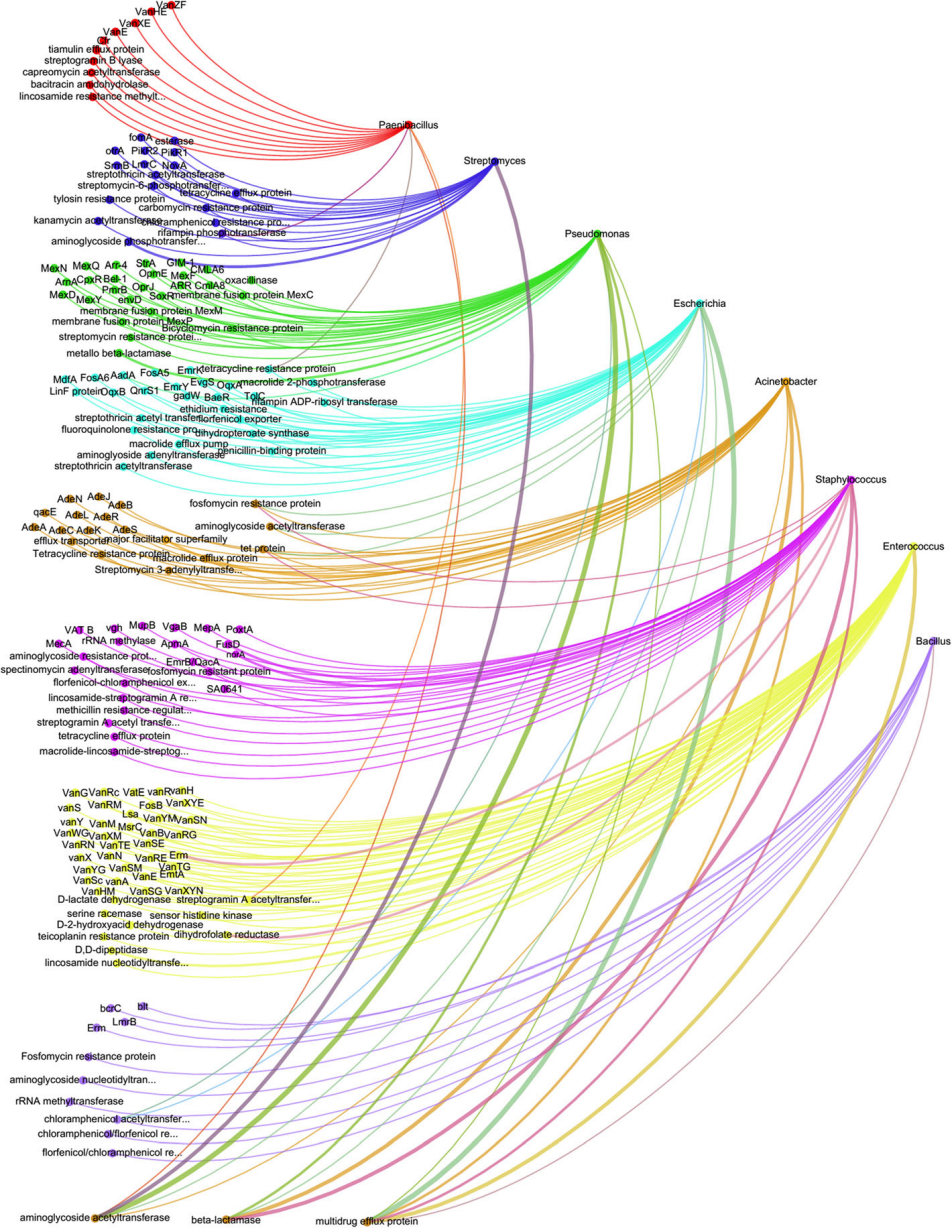
Network graph showing the correlation among the microbial genera and antibiotic resistance related genes. The graph represents only the positively correlated significant values (*P* < 0.05). Thick lines indicate relatively higher abundance

A total of 7442 and 7314 ORFs were aligned with 571 and 531 genes, respectively, in the Comprehensive Antibiotic Resistance Database (CARD) database. The BLASTx analysis against the CARD database indicated the presence of antibiotic-resistant genes associated with tetracycline, rifamycin, and vancomycin. The genes related to multidrug transporter protein and microbial pathogenicity (e.g., *Neisseria gonorrhoeae* MacB proteins) were also detected (Table S15 B). The correlation network graph showed the prevalent microbial genera, *Pseudomonas*, *Streptococcus*, *Staphylococcus*, *Streptomyces*, *Paenibacillus*, *Bacillus*, *Acinetobacter*, *Enterococcus*, and *Escherichia*, as the source of antibiotic-resistant genes in these thermal springs (Fig. 5; Table S16).

Further to the above, the metagenomic analysis depicted the presence of many genes that could be involved in the interspecies competition and microbial life survival in harsh conditions, for example, permeases of different facilitator protein families, signal transduction histidine kinases, outer membrane receptor proteins, and various peptidases and proteases that help the microbes to sense their surrounding environment and tackle the antimicrobial compounds secreted by other microbes for maintaining the population homogeneity in the habitat.

#### Carbohydrate metabolism

**Fig. 6:**
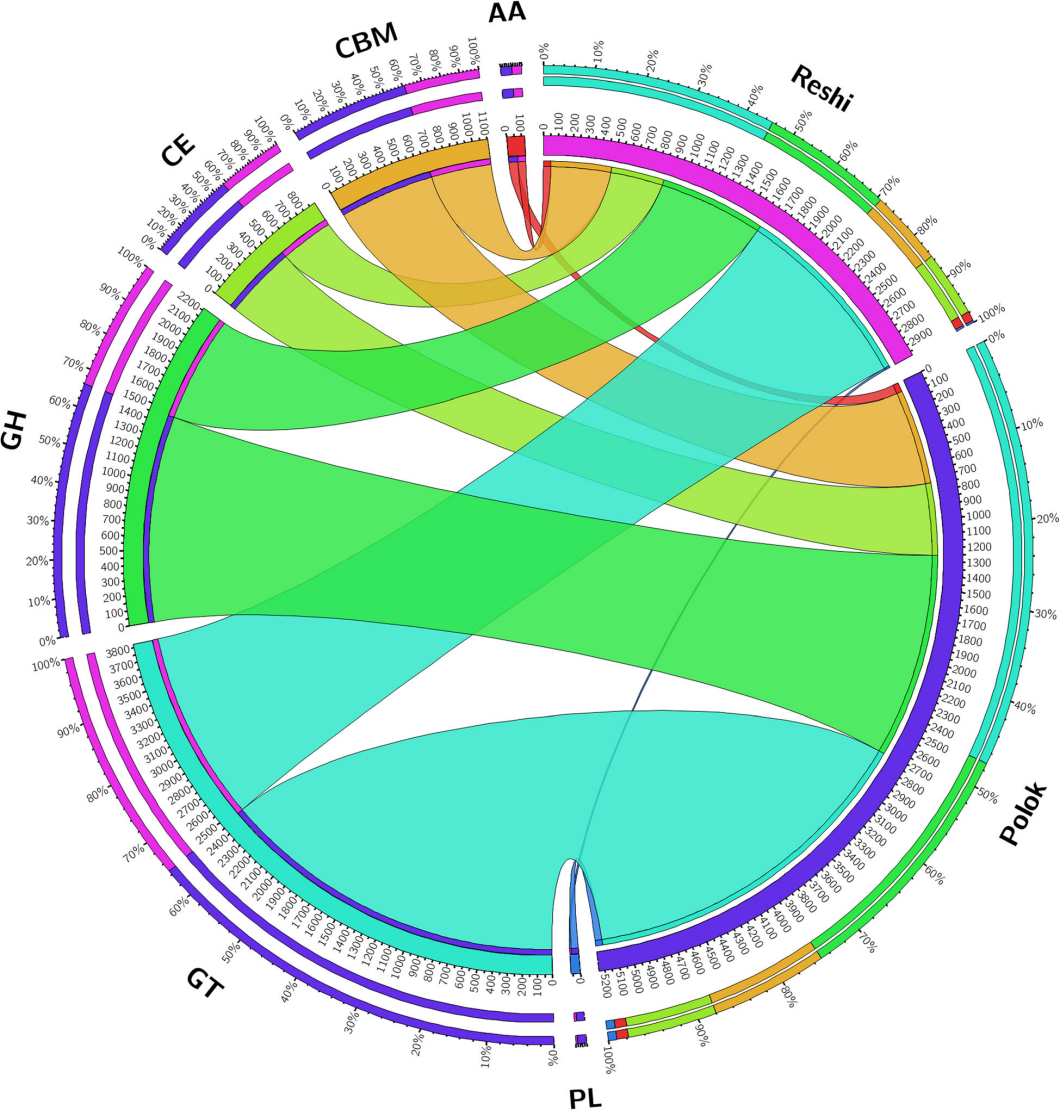
Circos figure showing the relative abundance of CAZymes in Polok and Reshi metagenome with minimum 30% difference at protein level. Supportive data to the figure is given in the S17

A total of 6730 and 3286 putative CAZymes were identified in Polok and Reshi hot springs, respectively. A total of 5238, and 2979 novel putative genes were found in Polok and Reshi metagenomes, respectively, exhibiting more than 30% difference at protein level from the known CAZymes in the public domain (Table 3). The putative genes depicted the association with different carbohydrate related enzymes- glycoside hydrolase (GH), polysaccharide lyase (PL), glycosyltransferases (GT), carbohydrate esterases (CE), auxiliary activity (AA), and carbohydrate-binding modules (CBMs) (Fig. 6). GT and GH were the most represented families in both the hot-springs (Table S17).

**Table 3:** Carbohydrate-Active enZYmes in the Polok and Reshi metagenomes

CAZy Families	Polok		Reshi	
	Total CAZymes	Novel putative CAZymes^**a**^	Total CAZymes	Novel putative CAZymes^**a**^
Glycosyl Transferases (GT)	2771	2471	1250	1351
Glycosyl Hydrolases (GH)	2275	1462	1124	754
Carbohydrate Binding Modules (CBM)	1049	675	515	430
Carbohydrate Esterases (CE)	388	514	260	377
Polysaccharide Lyases (PL)	140	50	43	12
Auxiliary activity enzymes (AA)	107	66	94	55
**Total count**	**6730**	**5238**	**3286**	**2979**

^a^Putative genes exhibiting at least 30% difference at protein level (70% subject coverage cutoff) from the orthologous genes in public domain

GT was the most abundant family, with 2471 and 1351 CAZymes in Polok and Reshi metagenomes, respectively. Under the class of GT, prevalent subclasses in both the hot springs were GT4 (924 in Polok, 492 in Reshi), GT2 (904 in Polok, 507 in Reshi) (Table S17). GT related genes, known to be critical in the acclimatization of the microorganisms in the harsh environment, were identified in the metagenome, e.g., amino acid adenylation proteins, aminotransferases, sugar phosphorylases, ABC-type transporters, capsular biosynthesis proteins, cell wall biosynthesis proteins, dolichol-phosphate sugar transferases, phosphodiesterase’s, transcription regulatory proteins, and methyltransferases. Among the glycoside hydrolases, GH0 (193), GH3 (109), and GH13 (81) in Polok, and GH0 (75), GH43 (67), and GH3 (48) CAZymes were in prevalence in Reshi. These enzymes are majorly involved in carbohydrate hydrolysis, e.g., galactosidases, amylases, pullulanases, glucosidases, etc. CAZymes belonging to Carbohydrate-Binding Module (CBM), which are known to accelerate the activity of other enzymes, were detected in Polok and Reshi hot springs, e.g., CBM50 (349 in Polok, and 225 in Reshi), CBM13 (81 in Polok, and 35 in Reshi), and CBM 48 (55 in Polok, and 46 in Reshi). The CBMs related to penicillin-binding, peptidoglycan binding, LysM, chitinases, and different trans-glycosylases involved in various metabolic functions were identified in both the metagenomes. Apart from this, genes related to deacetylases, acetyltransferases, and esterases were noted, e.g., CE11 (101 in Polok, and 80 in Reshi), CE13 (81 in Polok, and 35 in Reshi), and CE48 (55 in Polok, and 46 in Reshi). A few genes represented PL and AA class of enzymes related to the functions of laccases, pectate lyases, heparinases, proxidases, catalases, etc. (Table S17).

#### Statistical validation of functional profile

The diversity and relative abundance of orthologous genes in both the hot springs were statistically validated by the STAMP tool using Fisher exact test with Bonferroni correction. Top 100 COG functions in both the hot spring showed the diverse metabolic potential in both the hot springs (Fig. S9; Table S18). Iron transporters and transcription regulators were highly represented in Reshi hot spring, while Polok exhibited the prevalence of Trico peptide repeats (TPR) and molecular chaperones that help in maintaining stable protein structure at high temperature. The predicted genes for glycosyltransferases, permeases, and epimerases were relatively more in Polok.

## Discussion

Sikkim is a small state nestled in the lap of Himalaya, where nature has nurtured various moderate to high-temperature thermal springs, enshrined with discrete presence of flora, fauna, and microbial communities. Only a few Himalayan hot springs, located in the remote and harsh-environment prevailing conditions, have been explored for molecular profiling of taxonomic and metabolic features [9, 11, 13]. Polok and Reshi hot springs are located at the altitudinal difference of about 460 m in the vicinity of the greater Himalaya in South and West Sikkim, respectively. The alkaline condition in Polok and Reshi could be due to the presence of carbonates, bicarbonates, and hydroxide compounds of different metals ions viz., calcium, sodium, and potassium [13, 17]. The alkalinity and warmness properties of the geothermal spots are believed to exert therapeutic effects related to skin health, blood circulation, etc. [18, 19]. The molecular profiling is crucial to specify the genomic signatures of microfloral diversity and metabolic potential in an ecological niche [10].

The present whole-metagenomic investigation determines Reshi and Polok warm springs to be a precious resource of mesophiles and moderate thermophiles. The geothermal hot water spots of 25 to 70 °C temperature range have been recorded to be enriched with a wealthy microbiome [9, 10, 20, 21]. The abundance of Proteobacteria, Firmicutes, and Bacteroidetes in Polok and Reshi is in agreement with the previous reports on 16sRNA sequencing-based microbiota analysis of geothermal springs of the northeast region of India, and other parts of the world [9, 22–24]. Reshi, the aquatic habitat of slightly warm temperature (43 °C), braced relatively inflated Proteobacteria species diversity as compared to Polok hot spring (60 °C), as observed previously [25]. Alpha-proteobacteria and gamma-proteobacteria were the predominant class of Proteobacteria in the hot spring. Hot springs of moderate temperatures are supposed to inhabit thermophilic photosynthetic bacteria, such as Cyanobacteria and Chloroflexi [22, 25, 26]. The abundance of both the phyla is reported to be positively correlated in thermal springs [27]. However, in this study, the relatively reduced presence of cyanobacterial species was noted in Polok and Reshi, which was similar to the earlier studies on metagenomics of thermal aquatic habitats of this region [11, 28]. This signifies that the growth of cyanobacteria could be influenced by some other geochemical factors in these sites, which need to be further investigated. Furthermore, the genomic presence of Chloroflexi and Deinococcus-Thermus was found higher in Polok in comparison to Reshi, which could be due to the temperature difference (17 °C) of both the hot springs. Thus, Polok habitat, with the higher side of the temperature (60 °C), is suitable for supporting the growth of thermophiles or hyperthermophiles from Aquificae and Thermotogae. A similar observation has been made in a previous metagenomic investigation on the hot water sites of this temperature range [11, 28, 29].

Although Proteobacteria and Firmicutes phyla were predominant in both the hot springs, the difference in ecological features led to differential growth of microbial population belonging to the distinct classes and genera under these phyla. Beta-proteobacteria (*Massilia*, *Thiomonas*, and *Janthinobacteria*), detected only in Reshi hot spring, represent the peculiar mesophilic type of environment. These genera have also been reported from other hot water sites [30–32]. However, it cannot be ruled out that these mesophilic microorganisms could be from the soil. These mesophiles are known to produce enzymes to metabolize nitrogen, sulfur, and biosynthesize antimicrobial compounds [32, 33]. The positive correlation among nitrogen and sulfur utilizing Proteobacteria and Firmicutes, with Bacteroidetes, could be indicative of copiotropic-oligotrophic microbial associations in the warm aquatic habitats [34]. In Reshi ecosystem, the substantial presence of alpha-proteobacteria could indicate oligotrophic activities [35]. Similarly, the anaerobic bacterial community from Epsilon-proteobacteria and Delta-proteobacteria (*Desulfacinum*, *Desulfobacca* in Polok; *Sulfovorum*, and *Desulfatirhabdium* in Reshi) are reported to be involved in executing the biogeochemical sulfur cycles in hot water bodies [36, 37].

The sequences related to some psychrophiles were also discerned, e.g., *Paenisporosarcina* sp. (3.25% in Polok, and 0.77% in Reshi), and *Sporosarcina* sp. (1.01% in Polok and 0.95% in Reshi) in the warm aquatic habitat of Sikkim Himalaya. The presence of these psychrophilic microorganisms could be the result of natural interventions of halophilic alkaline water bodies surrounded by colder spots [38, 39]. However, the survival strategy of the low-temperature adapted bacteria in hot water habitats is poorly understood [39] and needs to be explored in further details. The differential physicochemical environmental factors in Polok and Reshi warm water habitats flourished distinct microbial diversity, as evident in the clustering of the species abundance represented in PCA plot analysis (Fig. S4), signifying discrete ecological characteristics at different altitude [11].

The metabolic potential analysis of metagenomic sequences of Polok and Reshi inferred gene homologs mapped to different metabolic pathways such as nitrification, sulfur-oxidation, methanogenesis, carbohydrate-active enzymes, and antimicrobial compounds, etc. The operation of these pathways is related to the utilization of chemical energy into the metabolic functions of the niche microflora [40]. The microorganisms have evolved the genetic potential to metabolize the abundant chemical compounds in the thermoaquatic ecosystems for survival purposes [41, 42].

The concentration of nitrogen, estimated in the form of nitrites, in Polok and Reshi hot water samples are about 1.8, and 3.4 mg/mL, respectively [11, 13]. Unearthing the genetic traces of the nitrate/ nitrite transporters in the Polok and Reshi metagenomes highlights the importance of nitrogen as terminal electron acceptors; however, at the same instant, lack of ammonia transporters infers the utilization of free ammonia as a nitrogen source [41]. Anaerobic metabolism in microbes enables the uptake of nitrogen, found in the form of nitrates and nitrites in the surrounding environment. Gene homologs representing the assimilatory and dissimilatory nitrate reduction pathways are crucial genetic signatures of active nitrogen metabolism in Polok and Reshi. Moreover, the microbial phyla, Proteobacteria, Nitrospirae, and Verrucomicrobia, detected in the metagenomic investigation, are known to participate in operating the biogeochemical nitrogen cycle [43].

Sulfur is a significant constituent dissolved in warm water environments. The water samples of both the hot spring sites are known to contain sulfur [11, 13]. The estimated Sulphur (SO_4_) concentration is 12.9, and 21.2 mg/mL in Polok and Reshi, respectively [11, 13]. Sulfur is associated with various benefactions related to skin-health, nutritional, as well as antimicrobial, and antiallergic effects, etc. [44, 45]. Sulfur is also involved in thermal stabilization of proteins via a disulfide bonding mechanism, which helps the microorganisms to thrive in the warm surroundings [46]. The occupancy of the thermophilic dissimilatory sulfate reducers, Bacteroidetes, Ignavibacteria, and Chlorobi, in the metagenome, strengthens the inference of metagenomic data [41]. Furthermore, abundant Proteobacteria, including delta and epsilonproteobacteria, are also known to be sulfate utilizers in warm water environments [11, 47].

Hot springs are reported to indwell methane-producing organisms [48]. Methanogenesis is a critical component of the carbon cycle of the Earth’s biosphere, which is generally executed by the archaeal community, especially when traditional electron acceptors (e.g., oxygen, nitrate, and sulfate) are exhausted in the ecosystem [49]. Three well-known pathways of methanogenesis are acetate to methane [50], CO_2_ to methane [51], and methanol to methane [52]. Archaea can utilize different carbon sources, such as acetate, formate, CO_2_, methanol, methylamines, and methyl-sulfides, to reduce them into methane gas [53]. Acetate is a preferable precursor compound used for the production of biogenic methane. But there are limited genera of methano-archaea, i.e., *Methanosarcina* and *Methanosaeta* (previously known as *Methanothrix*), that specifically use acetate for methanogenesis [54]. The metagenomes were noted to have sequences representing bathyarchaeota, which, apart from euryarchaeota, are reported to perform methanogenesis [49]*.* In an ecosystem, methanogenic archaea and methanotrophic bacteria, as detected in both the metagenomes, often exhibit symbiotic relationships to regulate the global carbon metabolism and carbon fixation. In biomethanation, methyl-coenzyme M reductase (MCR) is a crucial enzyme complex that catalyzes the conversion of 5-Methyl THSM(S) PT, obtained from formate or acetate, to methane. MCR is also involved in methanotrophic activities [49, 50]. The abundance of orthologous genes encoding MCR in Polok is consistent with the higher presence of methanogens perceived in Polok.

Antibiotics are microbicidal compounds, which are produced by the microbes to overcome the inter-specific competition. Many reports have emphasized the anti-infective potential of the microbial population residing in the geothermal springs [55]. Such antimicrobial biomolecules are streptomycin, vancomycin, cephalosporins, monobactam, etc. Many microbial species of Actinobacteria are known to produce these antimicrobial compounds in diverse habitats [56–58]. Streptomycin, in particular, belongs to the class aminoglycosides that can hamper the protein translation machinery, inhibiting microbial growth. The present study detected the prevalence of genes related to streptomycin biosynthesis. In the course of evolution, microbes have developed the ability to curb the inter-species competition by various methods, e.g., secretion of bacteriocines, antimicrobial peptides, and antibiotics [57, 59, 60]. Actinobacteria, lavished in both the niches, can be the primary producer of these antimicrobial compounds [61]. These gram-positive bacteria, equipped with high GC content genome, have been identified from diverse ecosystems [62]. They are known to release antimicrobial compounds in hot water habitats to curb interspecies stress [63]. The hot springs with the characteristic temperature, alkalinity, different metals, and sulfur compounds, accompanying the antimicrobial compounds, confer the possible health benefits to humanity, which need to be investigated in further molecular details.

This study propounds companionship of antibiotic inhibitors in the warm aquatic niches, such as beta-lactamases, which inhibit the broad-spectrum beta-lactam antibiotics like penicillin, cephalosporin, monobactam antibiotics, etc. In agreement with our findings, a recent study reported the evidence of antibiotic resistance-related genes in the metagenomic analysis of Reshi hot spring [64]. Furthermore, diverse environmental niches, including pristine habitats, such as glacial soils, lakes, and aquatic habitats, have been recorded to foster a basal level of antibiotic resistance globally [65–68]. The antibiotic resistance genes could be acquired by the residing microbes under unfavorable conditions by horizontal gene transfer and natural transformation mechanisms [69, 70]. The perils of antibiotic synthesis and resistance are yet to be studied in-depth in the ecological niche of the Himalayan region.

The metagenomic investigation revealed a considerable representation of carbohydrate-active enzymes, which could be involved in many crucial biological phenomena [71]. Many GTs work as permeases, facilitating the transport of various metal ions as well as inorganic compounds essential for the survival of the microorganisms [72]. GT and the two-component regulatory system build stimulus-response cell circuits for the microbial survival in harsh conditions by sensing their surroundings through phosphorylation and de-phosphorylation mechanisms [73]. The GTs are supposed to work in coordination with the regulatory proteins and help in cell communication and the adaptation of the microbe in the surrounding environment, and O-linked and N-linked glycosylation of the polysaccharides, essential for the bacterial cell wall biosynthesis [74]. GHs are important enzymes of microbial carbohydrate metabolism and weaponry system. Hydrolases catalyze the degradation of carbohydrate molecules, increasing their bioavailability for microbes. Amylases (mainly alpha-amylases), pullulanases, dextranases, glucosidases, beta-glucosidases, beta-galactosidases, mannosidase, and glucosaminidases, are involved in the depolymerization of polysaccharides or shortening of large oligosaccharide. Some sugar phosphorylases identified in the metagenome, such as sucrose phosphorylases, and Lytic trans-glycosylases and Chitinases type of enzymes, could be involved in the cleavage of glycosidic linkage between N-acetylmuramoyl and N-acetylglucosaminyl residues present in cell wall of bacteria, resulting in the formation of a 1, 6-anydromuramoyl. They are majorly responsible for generating space within the peptidoglycan fraction and its recycling [75]. The presence of genetic information about these crucial carbohydrate-acting enzymes makes the metagenomic resource of great significance to the exploration of the biocatalyst systems for biomass-processing.

## Conclusion

The present study reports the first in-depth whole-metagenomic profiling of two hot springs, located at a different altitude of the Himalayan region. The study presents a piece of comprehensive molecular information about the microbial ecology as well as the metabolic features of these sites. The study revealed the molecular evidence of a diverse microbial wealth in the hot springs, statistically validated by SHE and β-diversity analyses. The difference in temperature and altitude of the thermal aquatic niches was represented by a differential abundance of the microbial species, belonging to Proteobacteria, Firmicutes, Chloroflexi, and Deinococcus-Thermus phyla. The genomic traces of some psychrophiles in the thermoaquatic sites depicted the adaptive evolution of these organisms for both cold and hot environmental exposures. Metagenomic investigation illustrated the presence of methanogenic archaea and methanotrophic bacteria involved in the global carbon cycle and carbon fixation in the ecosystem. The metabolic potential study revealed orthologous genes mapped to nitrification, sulfur-oxidation, methanogenesis, carbohydrate metabolism, and antimicrobial compounds, etc. Many genes related to diverse carbohydrate related activities were identified. The glycosyltransferases associated with maintaining the structural integrity, cellular communication, as well as adaptation mechanism of the resident microbes in the harsh environmental condition were found abundant in hot springs. The genomic evidence related to antibiotic biosynthesis, as well as antibiotic resistance, is a matter of environmental concern in the ecological niches of the Himalayan region. The humongous metagenomic repository of the Himalayan hot springs can be further explored to understand the dynamics of the ecosystem. The genomic resource of many enzymes crucial for carbohydrate molecule modifications was identified, indicating these sites as salient bioresource of biocatalyst systems of societal importance.

## Methods

### Sample collection

The water samples were collected aseptically from two hot springs located at the altitudinal difference of about 460 m in the South and West, respectively, of the Sikkim region, named Polok, and Reshi. The altitude, latitude, and longitude coordinates were measured by using GPS receiver (GPSmap76CSx, Garmin, Korea). The temperature and pH of the sample collection sites were examined by the portable thermometer and pH meter. The sampling was done in triplicates. The samples were carried to the laboratory under cool and sterile conditions and stored at − 80 °C. The DNA extraction was performed immediately after the transportation of the samples to the laboratory, i.e., within 3 days of sample collection.

### Metagenomic DNA extraction

The water samples were filtered through nitrocellulose filters (with 0.2 μm cut-off) to collect all the cellular forms present in hot spring waters. The membrane was suspended into the phosphate buffer (pH 7) and homogenized. Metagenomic DNA in the sample was then extracted by using the MP Bio-medicals DNA™ isolation kit, following the manufacturer’s instructions. The integrity and quantity of the isolated DNA were examined by agarose gel electrophoresis and NanoDrop 2000™ (Thermo fisher Scientific™), respectively. The DNA samples in triplicate were pooled in equal quantity and subjected to metagenome sequencing.

### Whole metagenome sequencing

Sequencing libraries were prepared from 200 ng total DNA by using True Nano DNA library preparation kit™ (Illumina), as per the manufacturer’s instructions. Whole metagenome sequencing was performed on Illumina MiSeq 2500 platform, generating paired-end reads (PE) (2 × 150 bp). The adapter sequences were removed using a flexible read trimming tool for Illumina NGS data, Trimmomatic (v 0.36) [76].

### Metagenome assembly and ORF prediction

The high-quality pair-end sequences were assembled into scaffolds by employing the de-Bruijn graph-based tool, metaSPAdes (v 3.10), at the default parameters. The assembled scaffolds were screened by using PROkaryotic DYnamic programming Gene-finding ALgorithm (Prodigal) (v2.6.3) tool, which filters the sequences on the basis of gene structure prediction and translation initiation site recognition [77]. This reduces the possibility of false positives and predicts ORFs efficiently in the data.

### Taxonomic profiling and functional annotation

The ORFs obtained from the prodigal tool were imported to the Kaiju metagenome classifier tool for taxonomic assignment. Kaiju tool translated the contigs into six possible reading frames and matched them with the NR protein database following the greedy heuristic approach and Burrows-Wheeler transform (BWT) algorithm, as explained by [78]. The match length and match score cut-off were set as 11 and 65, respectively, and the maximum five mismatches were allowed during taxonomic annotation.

The sequences predicted as ORFs by Prodigal were subjected to functional analysis by employing the COGNIZER (v0.9b) tool on default parameters. COGNIZER is a comprehensive stand-alone framework, which uses COG, KEGG, Pfam, GO, and FIG databases to infer functional terms in the metagenomic dataset [79]. Moreover, the assembled sequences were subjected to BLASTx (dbv 4) against the non-redundant (NR) protein database at 10^− 5^ e-value cut-off.

### Statistical analysis of microbial diversity and functional profiling

The data generated was statistically validated using different tools. Stat graphics centurion XVII was used to find Pearson product-moment correlation at the phylum level with 1% abundance (Statpoint Technologies, Inc., USA). The Principle component analysis (PCA), Shannon-Wiener evenness index (SHE), and β-diversity of the species abundance were calculated by the PAST (V 3.25), a composite package of statistical software [80]. Statistical validation of the functional data (i.e., COG functions) was done by STAMP (V 2.1.3) by generating an extended bar plot by applying Fisher Exact test and Bonferroni correction [81]. The significance of the data was determined at *p*-values < 0.05, which indicated the statistically significant non-zero correlations at the 95% confidence level. The correlation matrix between the antibiotic resistance-related genes (identified from the CARD database) and the microbial genera were determined using Pearson product-moment correlation plot. The network graph of the positively correlated microorganisms and antibiotic resistance gene was generated using Gephi (V 0.9.2).

### Carbohydrate-active enZYmes

The predicted ORFs, obtained from Prodigal, were mapped to the Carbohydrate-Active enZYmes (CAZy) database [82] on a threshold e-value of 10^− 5^. CAZy is a specific database comprised of ~ 1, 50,000 sequences with the catalytic domains of different classes of enzymes involved in various metabolic pathways of microbes [83].

### Antibiotic resistome in the metagenome

To find the genes associated with antibiotic resistance in the metagenome, the predicted ORFs were mapped against the CARD database (V 3.0.7) under default parameters. The CARD database is a vast repository of the genes associated with antimicrobial resistance (AMR) [84].

## Supplementary information

**Additional file 1: Figure S1.** (A) Species count commonly and exclusively present in Polok and Reshi, (B) Total number of mesophiles, thermophiles and psychrophiles in Polok and Reshi. **Figure S2.** Pearson product moment correlation plot at phylum level. **Figure S3.** Relative abundance of microbial species in Polok and Reshi hot springs. **Figure S4.** SHE analysis of microbial species distribution in Polok and Reshi. **Figure S5.** Principle component analysis (PCA) at species level. **Figure S5.** Principle component analysis (PCA) at species level. **Figure S6.** Metagenomic genes mapped to nitrogen metabolism. **Figure S7.** Metagenomic genes mapped to Sulphur metabolism. **Figure S8.** Metagenomic genes mapped to beta-Lactam resistance pathway. **Figure S9.** ORFs mapped to top 100 COG functions in the metagenome. Differential proportion of ORFs is statistically validated by STAMP tool using Fisher exact test with Bonferroni correction. The details of ORF distribution are given in Table S17.

**Additional file 2 Table S1** Domain level classification taxonomic composition in Polok and Reshi. **Table S2** Phylum level classification of taxonomic composition in Polok and Reshi. **Table S3** Family level classification of taxonomic composition in Polok and Reshi. **Table S4** Genus level classification of taxonomic composition in Polok and Reshi. **Table S5** Species distribution in Polok and Reshi. **Table S6** Psychrophiles, mesophiles, and thermophiles in the metagenome. **Table S7** Pearson product moment correlation among the species detected in the metagenome. **Table S8** Principle Component analysis at species level. **Table S9** Metagenomic ORFs assigned to different COG categories. **Table S10** Metagenomic ORFs assigned to different KEGG Categories. **Table S11** ORFs associated with nitrogen regulating genes in the metagenome. **Table S12** ORFs associated with sulfur metabolism genes in the metagenome. **Table S13** ORFs associated with methane metabolism genes in the metagenome. **Table S14** Genes associated with antibiotic biosynthesis in the metagenome. **Table S15** ORFs associated with antibiotic resistance genes in the metagenome from KEGG and CARD database. **Table S16** Putative antibiotics resistance genes and their possible source microbial genera from CARD database. **Table S17** Different CAZymes in the metagenome. **Table S18** ORFs associated with COG functions.

## Data Availability

The high-quality sequence reads have been submitted to the NCBI SRA database under the accession numbers, SRR10067286 and SRR10067287.

## Abbreviations

ORF: Open Reading frame
CAZymes: Carbohydrate –Active enZYmes
NR: Non redundant
PRODIGAL: PROkaryotic DYnamic programming Gene-finding Algorithm
COG: Cluster of orthologous genes
KEGG: Kyoto encyclopedia of genes and genomes
GO: Gene ontology
Pfam: Protein families
CARD: Comprehensive antibiotics resistance database
AMR: Anti-microbial resistance
