# Supplementary material for: Metagenomics revealing molecular profiling of community structure and metabolic pathways in natural hot springs of the Sikkim Himalaya
Source: BMC Microbiol. 2020 Aug 10;20:246. doi: 10.1186/s12866-020-01923-3 (PMC7418396; doi:10.1186/s12866-020-01923-3)
Supplement: Supplementary file 1 — Additional file 1: Figure S1. (A) Species count commonly and exclusively present in Polok and Reshi, (B) Total number of mesophiles, thermophiles and psychrophiles in Polok and Reshi. Figure S2. Pearson product moment correlation plot at phylum level. Figure S3. Relative abundance of microbial species in Polok and Reshi hot springs. Figure S4. SHE analysis of microbial species distribution in Polok and Reshi. Figure S5. Principle component analysis (PCA) at species level. Figure S5. Principle component analysis (PCA) at species level. Figure S6. Metagenomic genes mapped to nitrogen metabolism. Figure S7. Metagenomic genes mapped to Sulphur metabolism. Figure S8. Metagenomic genes mapped to beta-Lactam resistance pathway. Figure S9. ORFs mapped to top 100 COG functions in the metagenome. Differential proportion of ORFs is statistically validated by STAMP tool using Fisher exact test with Bonferroni correction. The details of ORF distribution are given in Table S17. [file 12866_2020_1923_MOESM1_ESM.pdf]

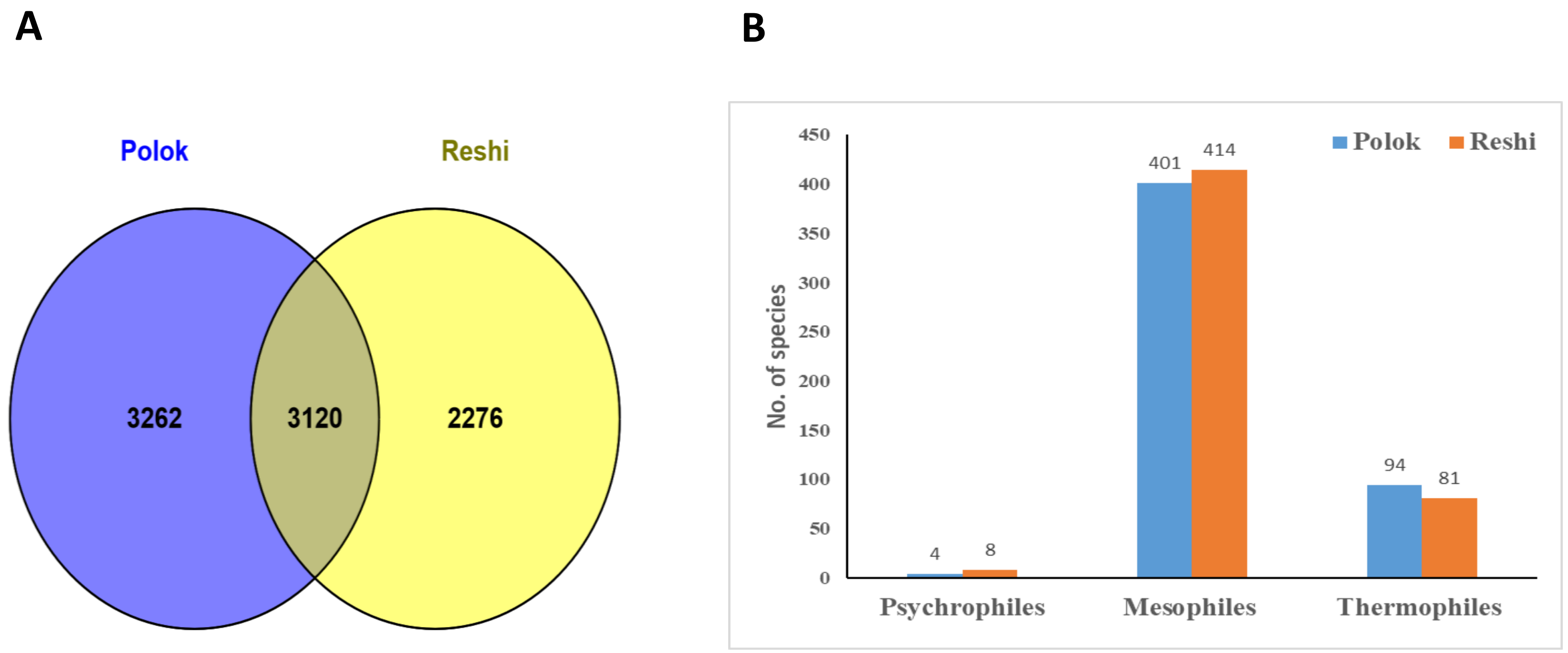

Fig. S1. (A) Species count commonly and exclusively present in Polok and Reshi, (B) Total number of mesophiles, thermophiles and psychrophiles in Polok and Reshi.

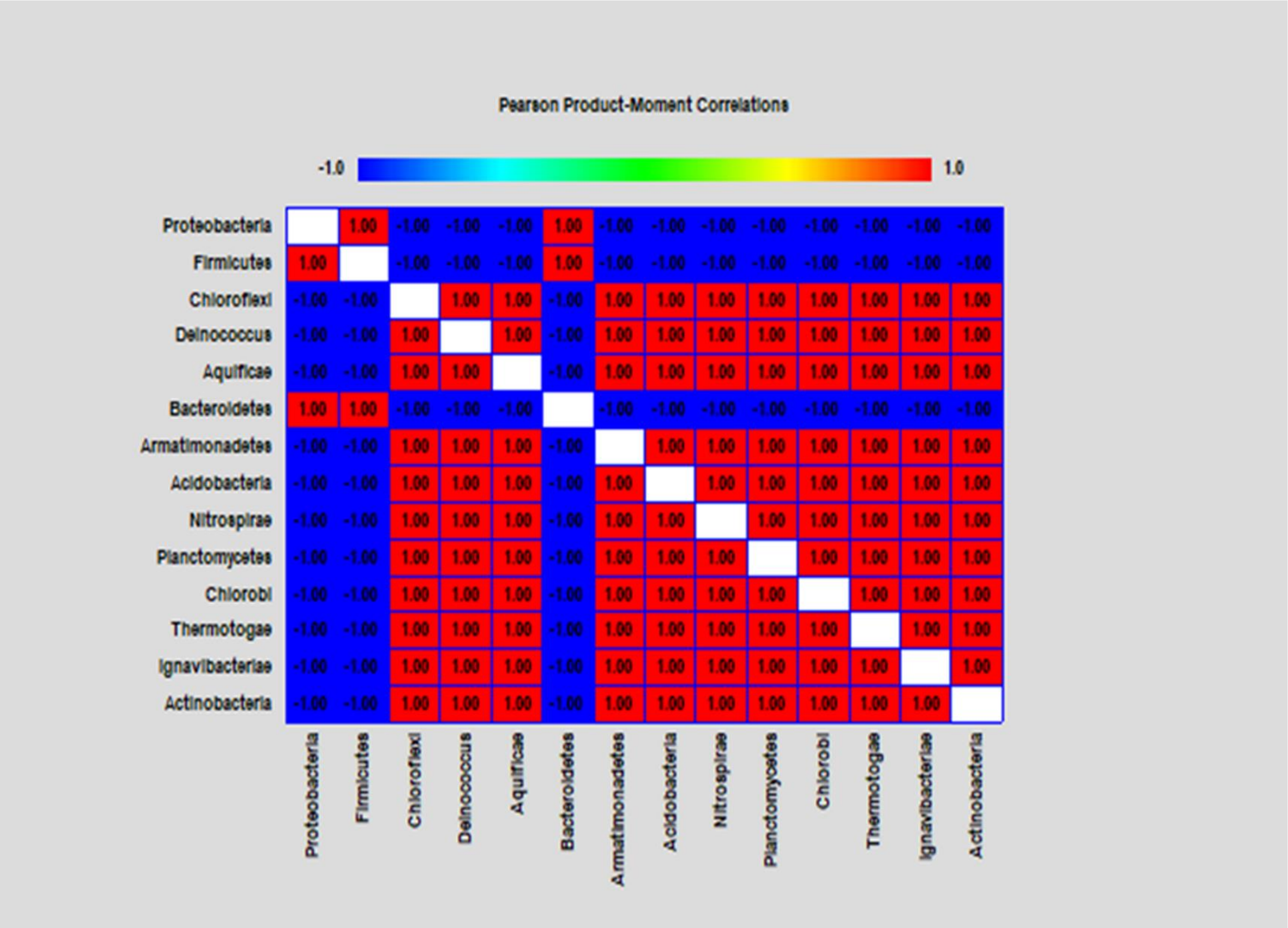

Fig. S2. Pearson product moment correlation plot at phylum level.

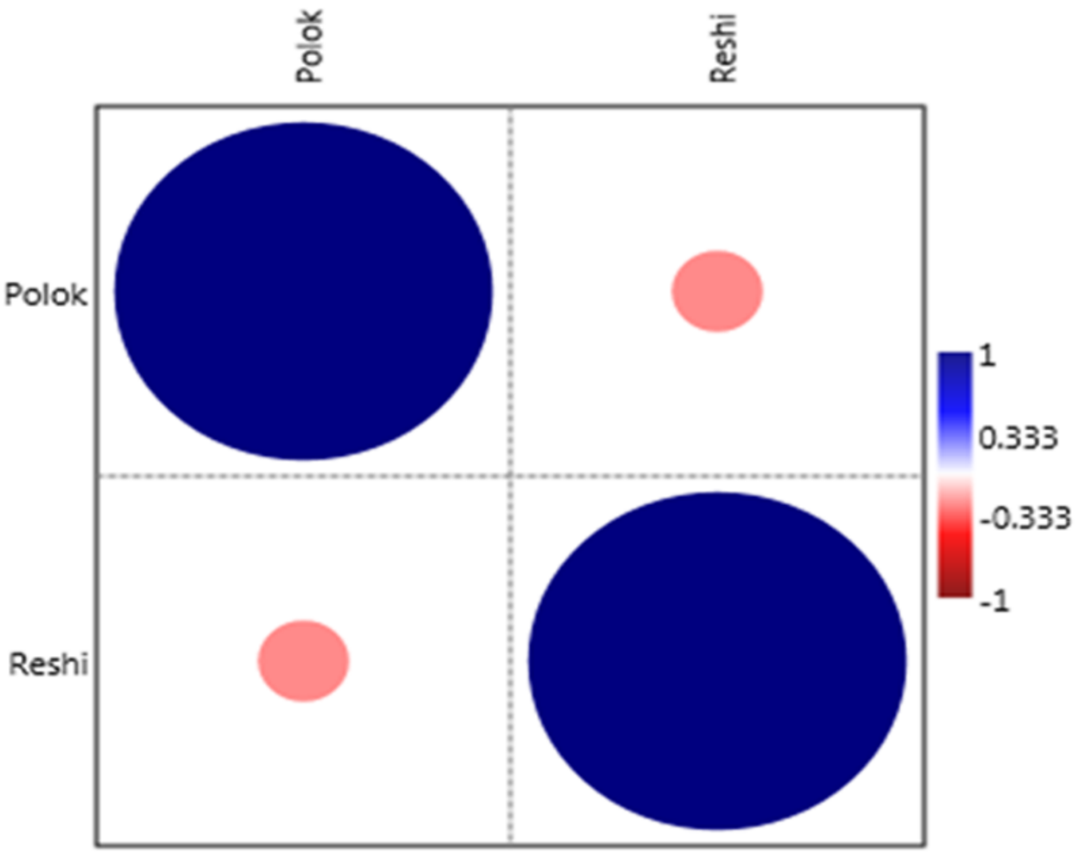

Fig. S3. Relative abundance of microbial species in Polok and Reshi hot springs.

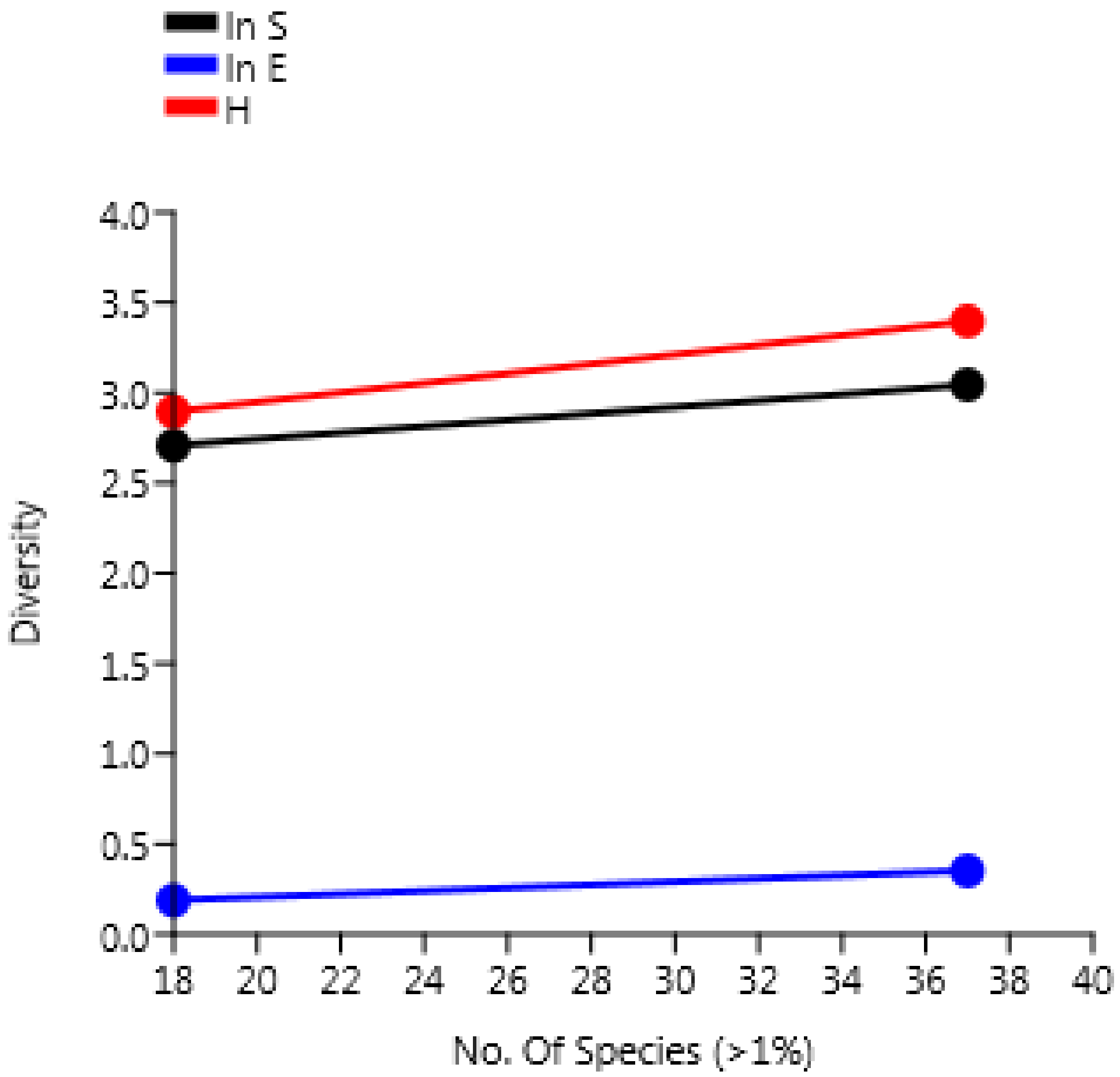

Fig. S4. SHE analysis of microbial species distribution in Polok and Reshi.

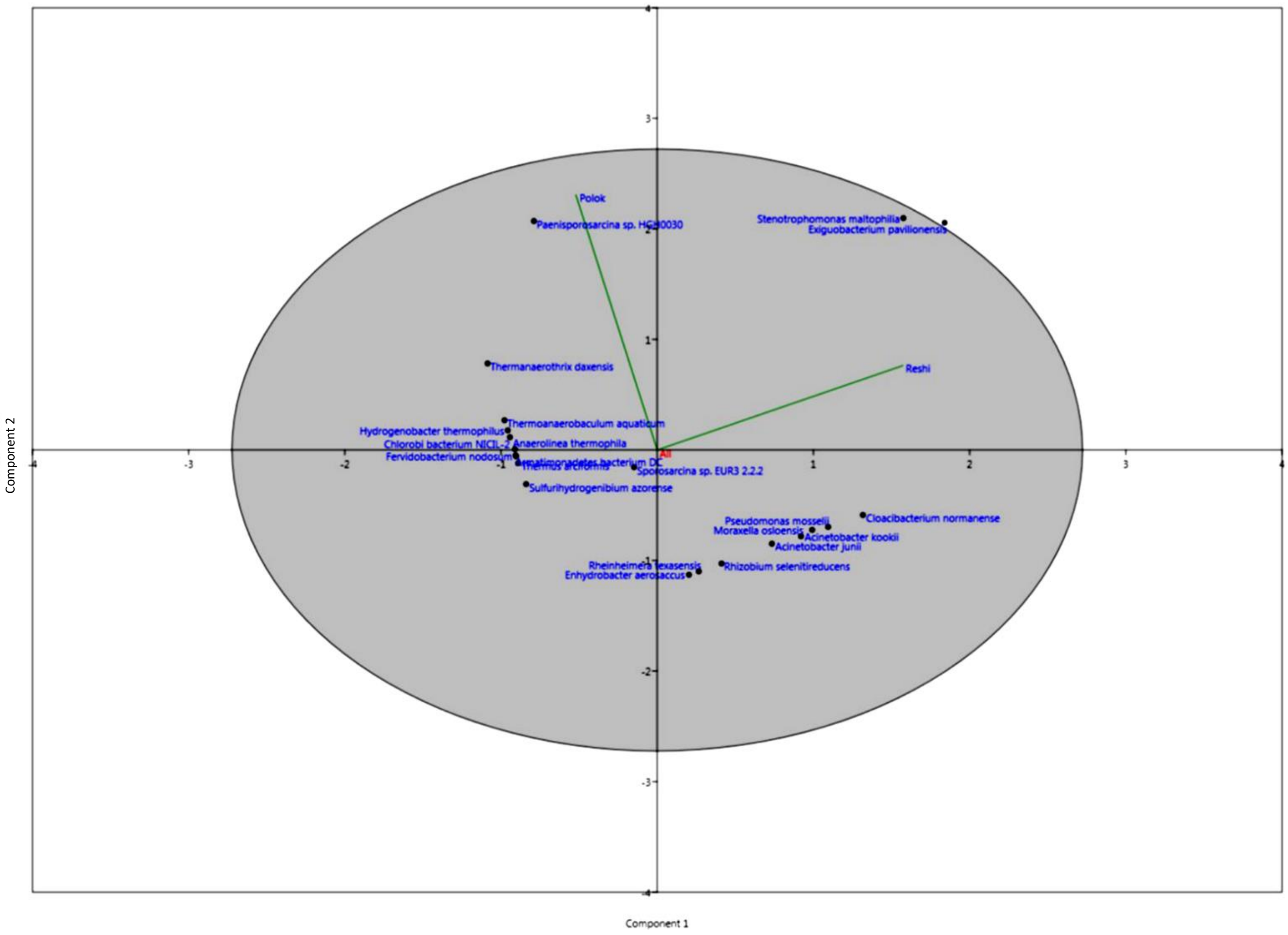

Fig. S5. Principle component analysis (PCA) at species level.

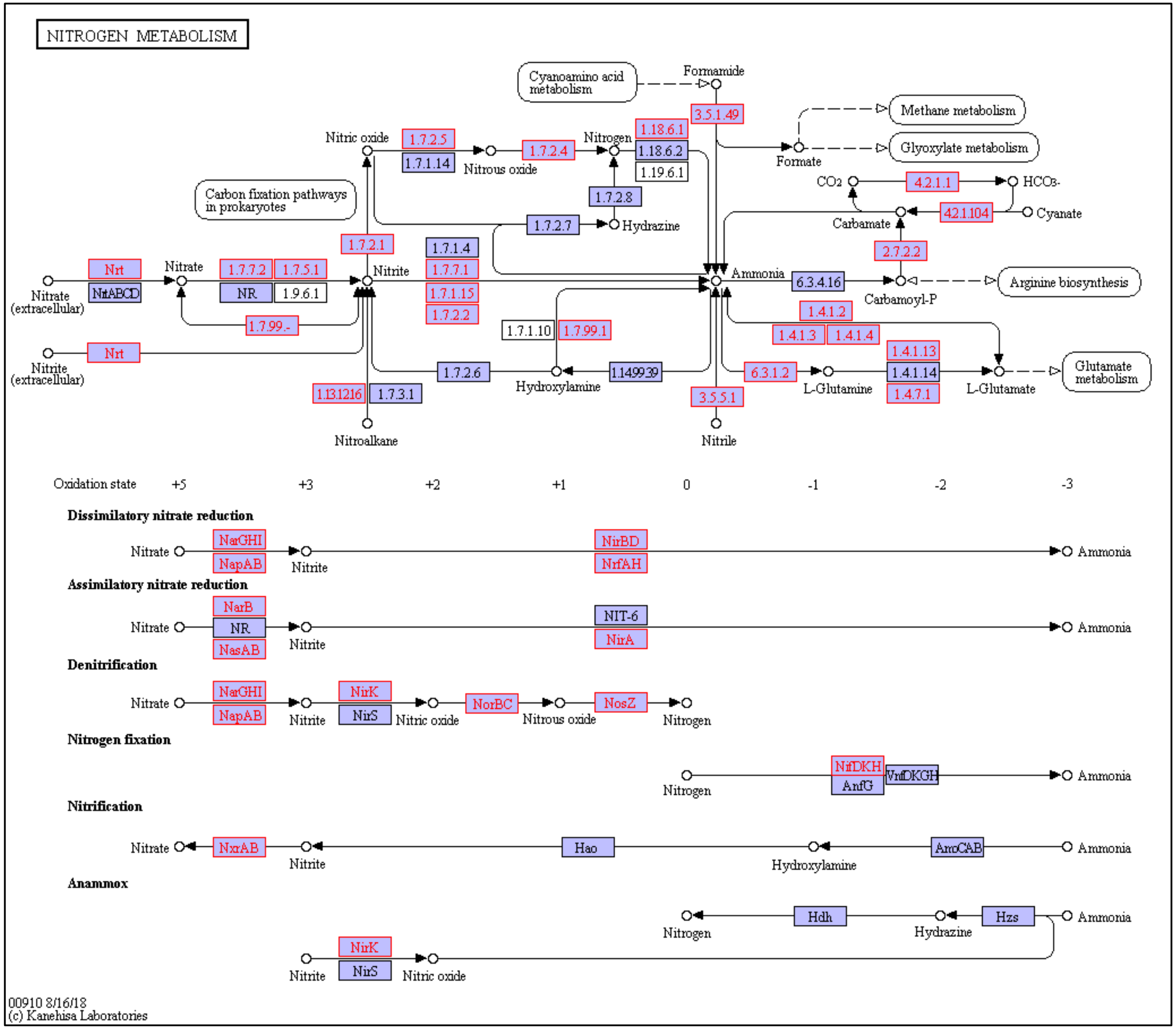

Fig. S6. Metagenomic genes mapped to nitrogen metabolism.

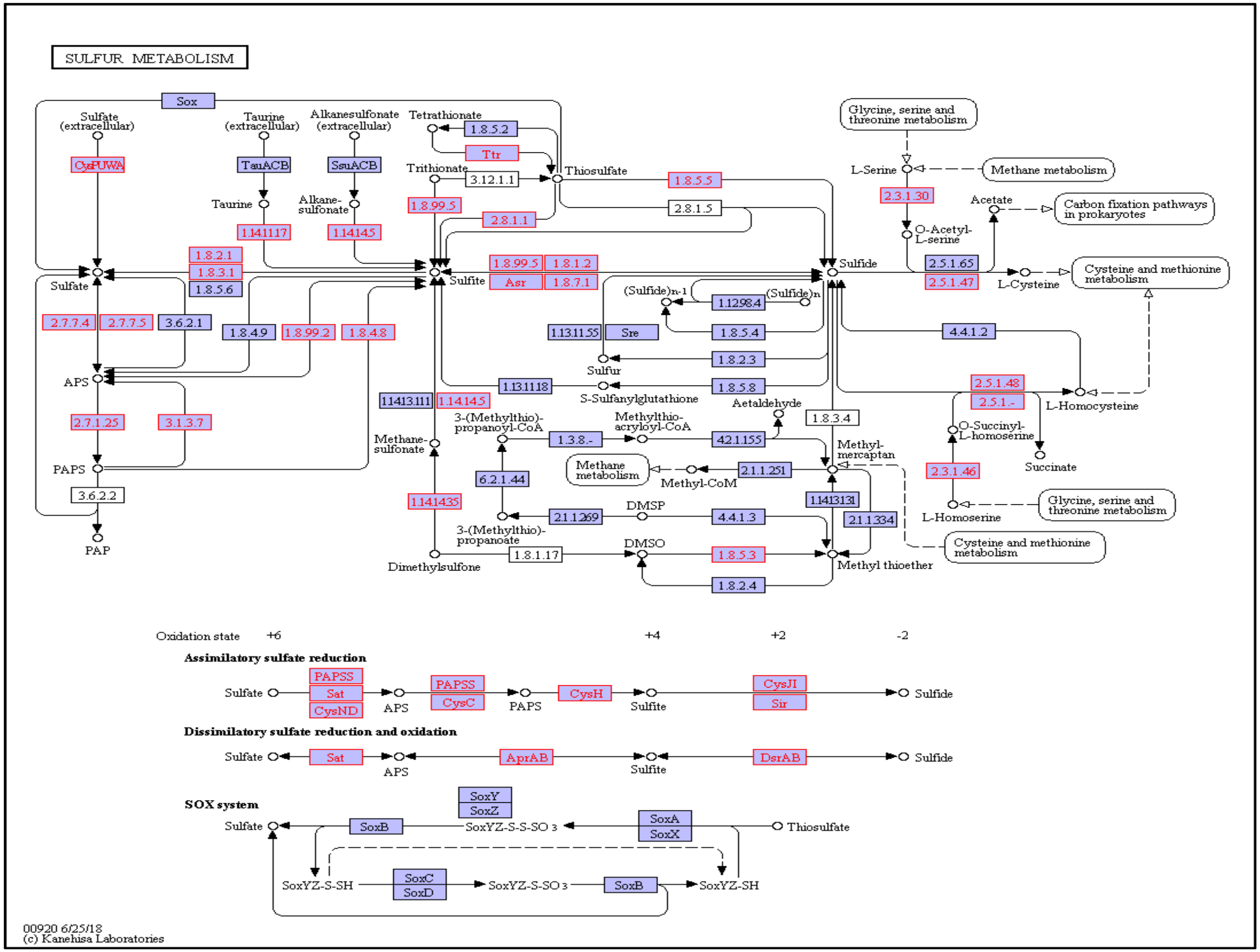

Fig. S7. Metagenomic genes mapped to sulphur metabolism.

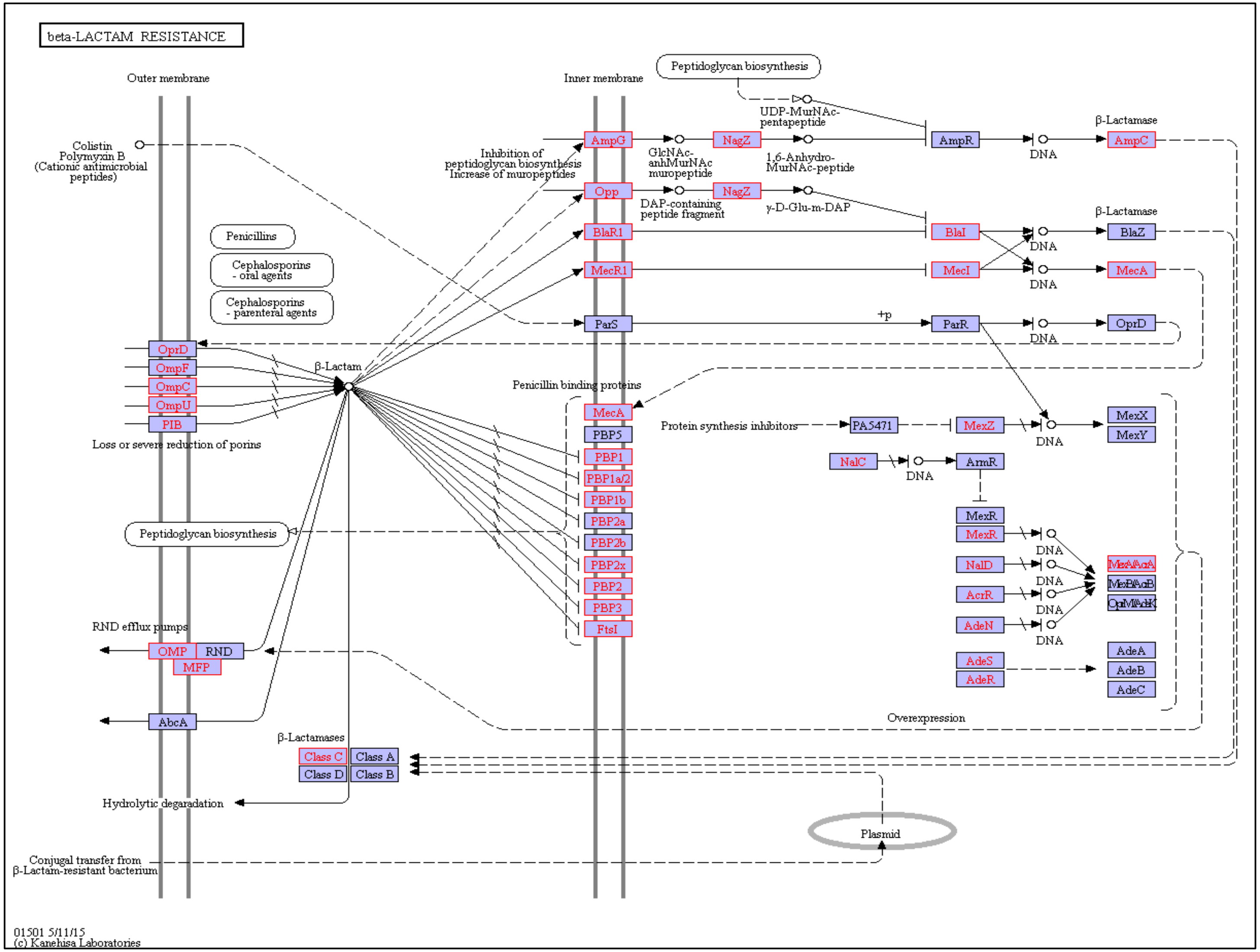

Fig. S8. Metagenomic genes mapped to beta-Lactam resistance pathway.

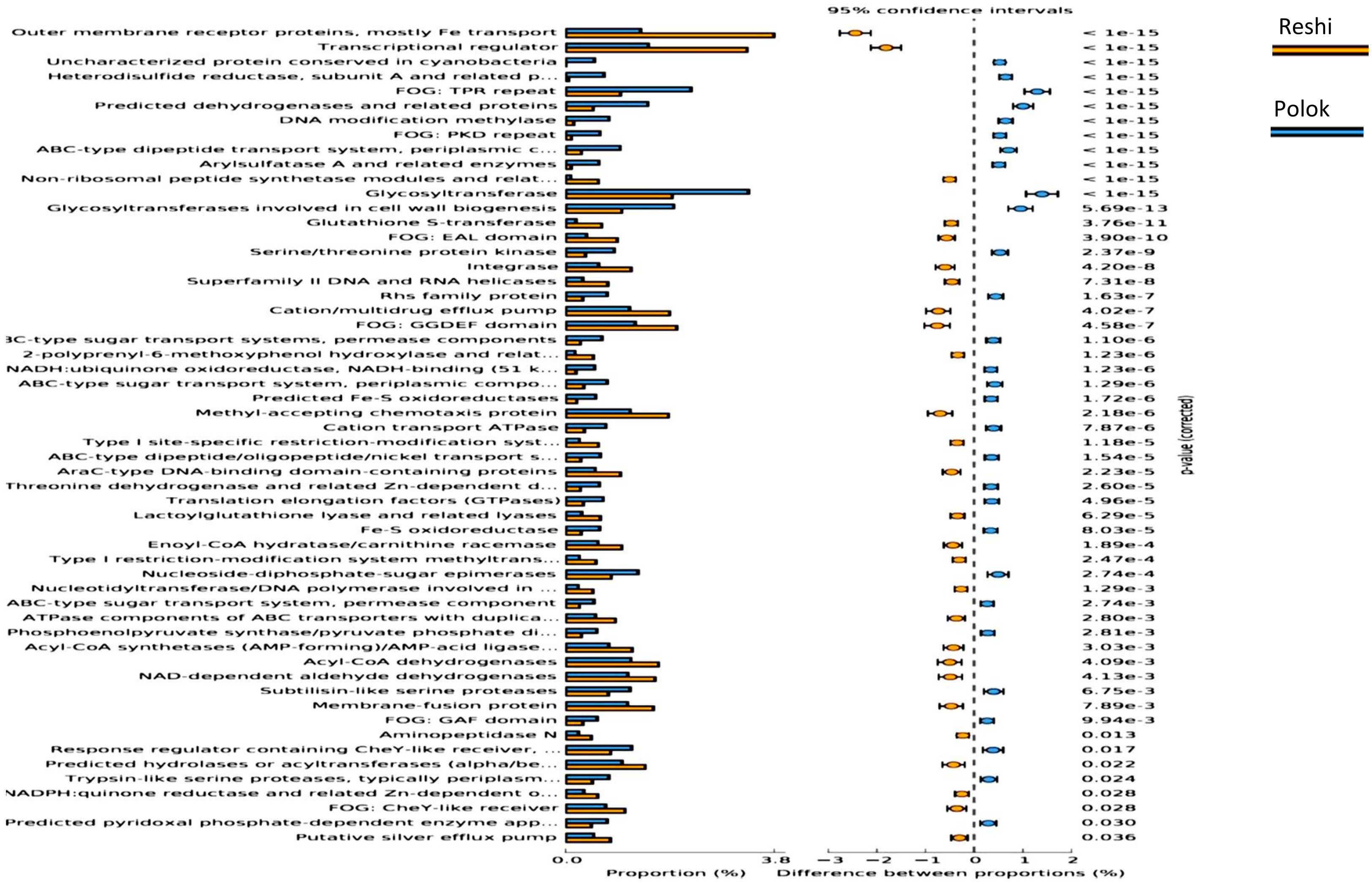

Fig. S9. ORFs mapped to top 100 COG functions in the metagenome. Differential proportion of ORFs is statistically validated by STAMP tool using Fisher exact test with Bonferroni correction. The details of ORF distribution are given in Table S17.
